# Prenatal and Perinatal Factors of Life’s Essential 8 Cardiovascular Health Trajectories

**DOI:** 10.1001/jamanetworkopen.2025.7774

**Published:** 2025-04-29

**Authors:** Izzuddin M. Aris, Sheryl L. Rifas-Shiman, Sarah D. de Ferranti, Marie-France Hivert, Wei Perng

**Affiliations:** 1 Department of Population Medicine, Harvard Medical School and Harvard Pilgrim Health Care Institute, Boston, Massachusetts; 2 Department of Pediatrics, Harvard Medical School, Boston, Massachusetts; 3 Diabetes Unit, Massachusetts General Hospital, Boston; 4 Department of Epidemiology, Colorado School of Public Health, University of Colorado Anschutz Medical Campus, Aurora; 5 Lifecourse Epidemiology of Adiposity and Diabetes (LEAD) Center and the Department of Epidemiology, University of Colorado Anschutz Medical Campus, Aurora

## Abstract

**Importance:**

The American Heart Association put forth the Life’s Essential 8 construct to assess cardiovascular health (CVH) based on 8 biological and behavioral factors. Few studies have identified prenatal and perinatal factors of CVH trajectories across childhood and adolescence, life stages where disease precursors and health behaviors are established.

**Objective:**

To examine associations of prenatal and perinatal factors with child CVH trajectory.

**Design, setting, and participants:**

Data from the Project Viva prebirth cohort from April 1999 to August 2021 were used. Participant inclusion required 3 or more CVH metrics in early childhood (median [range] age, 3.2 [2.8-6.2] years) or 4 or more in midchildhood (median [range] age, 7.7 [6.6-10.9] years), early adolescence (median [range] age, 13.0 [11.9-16.6] years), or late adolescence (median [range] age, 17.5 [15.4-20.1] years). Data were analyzed from April 1 to September 30, 2024.

**Exposures:**

Prenatal and perinatal factors.

**Main outcomes and measures:**

CVH score (0-100 points), calculated as the unweighted average of all available CVH metrics at each life stage.

**Results:**

Among 1333 children included, 680 (51.0%) were male, 78 (5.9%) Hispanic, 181 (13.6%) non-Hispanic Black, and 959 (71.9%) non-Hispanic White. The estimated mean (SD) age of inflection when CVH started to decline was 10.2 (0.7) years for male children and 10.0 (0.6) years for female children. Prepregnancy overweight or obesity (vs healthy or underweight), smoking during pregnancy (vs never), and formula-feeding (vs breastfeeding) in the first 6 months were each associated with lower CVH from childhood to adolescence, but gestational diabetes (vs normal glucose tolerance) was not associated with CVH. Prepregnancy obesity was associated with later inflection (β = 0.1; 95% CI, 0.0 to 0.2 years) and slower CVH decline after inflection (β = 0.2; 95% CI, 0.1 to 0.4 points per year). Gestational hypertension or preeclampsia (vs normal blood pressure) was associated with faster CVH gain before inflection (β = 0.3; 95% CI, 0.1 to 0.5 points per year), earlier inflection (β = −0.1; 95% CI, −0.2 to 0.0 years), and faster CVH decline after inflection (β = −0.3; 95% CI, −0.5 to −0.1 points per year), while smoking during pregnancy was associated with later inflection (β = 0.2; 95% CI, 0.1 to 0.3 years).

**Conclusions and Relevance:**

In this cohort study, prepregnancy overweight or obesity, smoking during pregnancy, and formula-feeding in the first 6 months of life were each associated with adverse CVH trajectories early in life. Future work should examine whether interventions that address these factors would be effective in optimizing CVH in children.

## Key Points

**Question:**

What are the prenatal and perinatal factors of Life’s Essential 8 cardiovascular health (CVH) trajectories across childhood and adolescence?

**Findings:**

This cohort study of 1333 children found that prepregnancy obesity, smoking during pregnancy, and formula-feeding in the first 6 months of life were each associated with persistently lower CVH by 1 to 2 points from childhood to adolescence, and statistically significant (but small) differences in CVH trajectory parameters including timing and rate of decline in CVH.

**Meaning:**

These findings suggest that future work should examine whether early-life interventions that address these factors would be effective in optimizing CVH in children.

## Teaser

This cohort study evaluates associations of prenatal and perinatal factors with offspring cardiovascular health from childhood to late adolescence.

## Introduction

Cardiovascular disease (CVD) is a leading cause of mortality in the US.^1^ Recent findings showed that childhood CVD precursors (ie, high body mass index [BMI], blood pressure [BP], and cholesterol) as early as age 3 years were strong predictors of CVD-related mortality in adulthood,^2^ emphasizing the importance of primordial prevention. The American Heart Association provides guidance on this issue via a cardiovascular health (CVH) construct known as Life’s Essential 8 (LE8), including 4 behavioral (nicotine exposure, diet, physical activity, and sleep duration) and 4 health factors (BMI, BP, glucose, and cholesterol, hereafter referred to as biological CVH) on a 0- to 100-point scale.^3^ However, prior studies showed that less than 2% of US children have optimal CVH scores of 100 points,^4,5^ and our recent study reported that children’s CVH began to decline at approximately age 10 years.^6^ As having higher CVH from childhood onwards confers lower risks of CVD and premature mortality^7,8,9,10,11^ and better quality of life in adulthood,^12^ promoting CVH from the beginning of life, even before birth, is of paramount public health importance.

Prior studies, including ours,^13,14,15,16,17,18,19,20^ have found associations of gestational exposures (eg, prepregnancy obesity, excessive gestational weight gain, and gestational diabetes) and behaviors (eg, prenatal smoking and breastfeeding) with CVD precursors, such as higher BMI and BP, from childhood to adolescence. Furthermore, 3 recent studies from the US and other countries reported that higher maternal BMI, BP, and glucose levels during pregnancy were each associated with lower overall CVH score and/or lower odds of ideal CVH in children^21,22^ and adolescents^23^ separately. Despite these observations, little remains known on how prenatal and perinatal factors shape the natural history of CVH across childhood and adolescence. A better understanding of these relationships is crucial to understanding whether there are sustained associations of the in-utero environment on CVH across early life which, in turn, could inform early interventions to avert the loss of and preserve CVH across development.

To address these research gaps, we used data from a prebirth cohort in eastern Massachusetts to examine associations of prenatal and perinatal factors with child CVH trajectory. We hypothesized that known risk factors of childhood CVD precursors, such as prepregnancy obesity, excessive gestational weight gain, gestational glucose intolerance, hypertensive disorders of pregnancy, smoking during pregnancy, and formula feeding in the first 6 months, would be associated with lower CVH from childhood to adolescence, as well as earlier and faster decline in CVH.

## Methods

### Study Population

We studied participants in Project Viva, an ongoing prebirth cohort study in eastern Massachusetts.^24^ From April 1999 to July 2002, trained staff recruited pregnant women attending their first prenatal visit. In-person visits with mother-child dyads occurred in infancy (median [range] age, 0.6 [0.4-0.8] years), early childhood (median [range] age, 3.2 [2.8-6.2] years), midchildhood (median [range] age, 7.7 [6.6-10.9] years), early adolescence (median [range] age, 13.0 [11.9-16.6] years), and late adolescence (median [range] age, 17.5 [15.4-20.1] years). We assessed up to 6 CVH metrics in early childhood and up to 8 from midchildhood to late adolescence. We included data from April 1999 to August 2021 on 1333 children (out of 2128 births) (eFigure 1 in Supplement 1) with information on prenatal and perinatal factors and at least half of CVH metrics at each life stage (ie, ≥3 in early childhood or ≥4 in midchildhood, early adolescence, or late adolescence). We used this approach to maximize our analytic sample, as the LE8 allows for generation of CVH scores regardless of the number of available metrics at each life stage.^3,4^ At each visit, we obtained written informed consent from mothers and either verbal assent (if aged <18 years) or signed consent (if aged ≥18 years) from the child. The Harvard Pilgrim Health Care institutional review board approved the study, which followed the Strengthening the Reporting of Observational Studies in Epidemiology (STROBE) reporting guideline for cohorts.

### Prenatal and Perinatal Factors

We selected 7 modifiable prenatal and perinatal factors based on previous Project Viva studies as well as prior systematic reviews and meta-analyses that have shown associations between each known risk factor and child CVD precursors.^13,14,15,16,17,18,19,20^ To facilitate clinical interpretability, we categorized all prenatal and perinatal factors using clinically relevant definitions.

Mothers reported their prepregnancy weight and height via questionnaires at enrollment, from which we calculated prepregnancy BMI (calculated as weight in kilograms divided by height in meters squared) and categorized as healthy or underweight (<25), overweight (25 to <30), or obesity (≥30). We determined total gestational weight gain (GWG) as the difference between self-reported prepregnancy weight and the last clinically measured weight recorded before delivery. We categorized GWG as inadequate, adequate, or excessive based on Institute of Medicine 2009 guidelines, which vary by prepregnancy BMI.^25^ We obtained results of a 2-stage clinical glycemic screening and used them to categorize women as having normal glucose tolerance, isolated hyperglycemia, impaired glucose tolerance, or gestational diabetes (GD), based on criteria previously detailed.^20^ We extracted data on hypertensive disorders of pregnancy (normal blood pressure, gestational hypertension or preeclampsia, or chronic hypertension) from medical records.

Mothers reported their smoking history via questionnaires at enrollment, which we categorized as never, before pregnancy, or during pregnancy. We obtained information on breastfeeding initiation (yes or no) at postdelivery interviews.^26^ At the infancy visit, mothers reported on infant feeding type in the first 6 months using self-administered questionnaires, which we grouped into 4 categories (breastfeeding only, weaned, mixed, or formula only) on the basis of their extent of breastfeeding or formula feeding as previously detailed.^18^

### Cardiovascular Health (CVH) Score

Project Viva collected information on up to 6 (out of the 8) CVH metrics (diet, nicotine exposure, physical activity, sleep duration, BMI, and BP) in early childhood, and up to 8 from midchildhood to late adolescence.^6^ We provide details on the assessment of each metric in the eMethods in Supplement 1. For each metric, we applied the LE8 scoring algorithm to calculate CVH scores (0-100 points) from early childhood to late adolescence based on current guidelines (eTable 1 in Supplement 1).^3,4^ We calculated an overall CVH score as the unweighted average of all available metrics at each life stage and calculated separate scores for behavioral and biological factors (see eMethods in Supplement 1).

### Covariates

We obtained self-reported information on maternal sociodemographic characteristics via interviewer-administered questionnaires at enrollment and operationalized them as follows: age (years), education level (noncollege or college degree and above), annual household income (≤$70 000 per year or >$70 000 per year), and race and ethnicity (Hispanic, non-Hispanic Asian, non-Hispanic Black, non-Hispanic White, or non-Hispanic Other [ie, American Indian, Alaska Native, Pacific Islander, or Other race (unspecified)]). Race and ethnicity data were reported because we considered those characteristics to be proxy measures of structural racism that can have implications for both exposure to risk factors of childhood CVD precursors and subsequent development of cardiovascular risk. We extracted data on parity (nulliparous or primiparous), infant sex (male or female), and birth weight from hospital medical records. We calculated gestational age (GA) by subtracting the date of the last menstrual period from the date of delivery. We calculated birth weight-for-GA *z* scores from a US national reference.^27^

### Statistical Analysis

We decided a priori to characterize sex-specific trajectories of CVH given the well-documented sex differences in CVD and its precursors.^20,28^ We used segmented mixed-effect models^29,30^ to characterize sex-specific trajectories of overall, behavioral, and biological CVH score from early childhood to late adolescence. This innovative extension of the piecewise mixed-model framework uses a data-driven approach to identify participant-specific inflection point(s) along the trajectory when the rate of change in CVH score is accelerating or decelerating. We described details of the model in the eMethods in Supplement 1. We used this model to estimate the following sex- and participant-specific trajectory parameters: (1) slope of CVH before the inflection point; (2) timing of inflection when CVH begins to accelerate or decelerate; (3) slope of CVH after inflection; and (4) projected CVH scores at ages 3 (reflective of the early childhood period), 8 (midchildhood), 13 (early adolescence), and 18 (late adolescence) years. We used linear regression models to estimate associations of each prenatal and perinatal factor with each CVH trajectory parameter and projected CVH scores from 3 to 18 years. We used a directed acyclic graph (eFigure 2 in Supplement 1) to inform covariate adjustment in our regression models.

We used chained equation multiple imputation to impute values for missing covariates. We generated 50 imputed datasets for all 2128 children. The imputation model included all prenatal and perinatal factors, CVH trajectory parameter outcomes, and covariates under study. We combined imputed datasets using Rubin rules^31^ with the MI ESTIMATE function in Stata after excluding the 795 children who did not satisfy the inclusion criteria. To assess the robustness of our findings, we conducted sensitivity analyses by repeating all analyses in 1079 children who had all available CVH metrics at each life stage. This approach ensures that at each life stage, all children would contribute the same number of CVH metrics toward the calculation of CVH score (ie, 6 in early childhood or 8 in midchildhood, early adolescence, or late adolescence). When interpreting findings, we focused on the direction, strength, and precision of the estimates and used a 2-sided α = .05 to assess statistical significance. We performed all analyses using Stata 16 (StataCorp) and R version 4.4.0 (R Project for Statistical Computing) from April 1 to September 30, 2024.

## Results

### Participant Characteristics

Of 1333 children included, 680 (51.0%) were male, 78 (5.9%) Hispanic, 181 (13.6%) non-Hispanic Black, and 959 (71.9%) non-Hispanic White. Compared with children included in the study, those excluded were more likely to identify as Black or Hispanic and less likely to have mothers with a college degree and household income higher than $70 000 per year (eTable 2 in Supplement 1). The mean (SD) CVH score was 82.7 (8.4) in early childhood, 84.3 (8.2) in midchildhood, 82.2 (9.7) in early adolescence, and 74.2 (11.4) in late adolescence. The estimated mean (SD) age of inflection when overall CVH started to decline was 10.2 (0.7) years for male children and 10.0 (0.6) years for female children (eTable 3 in Supplement 1).

### Overall CVH

**Figure 1.**
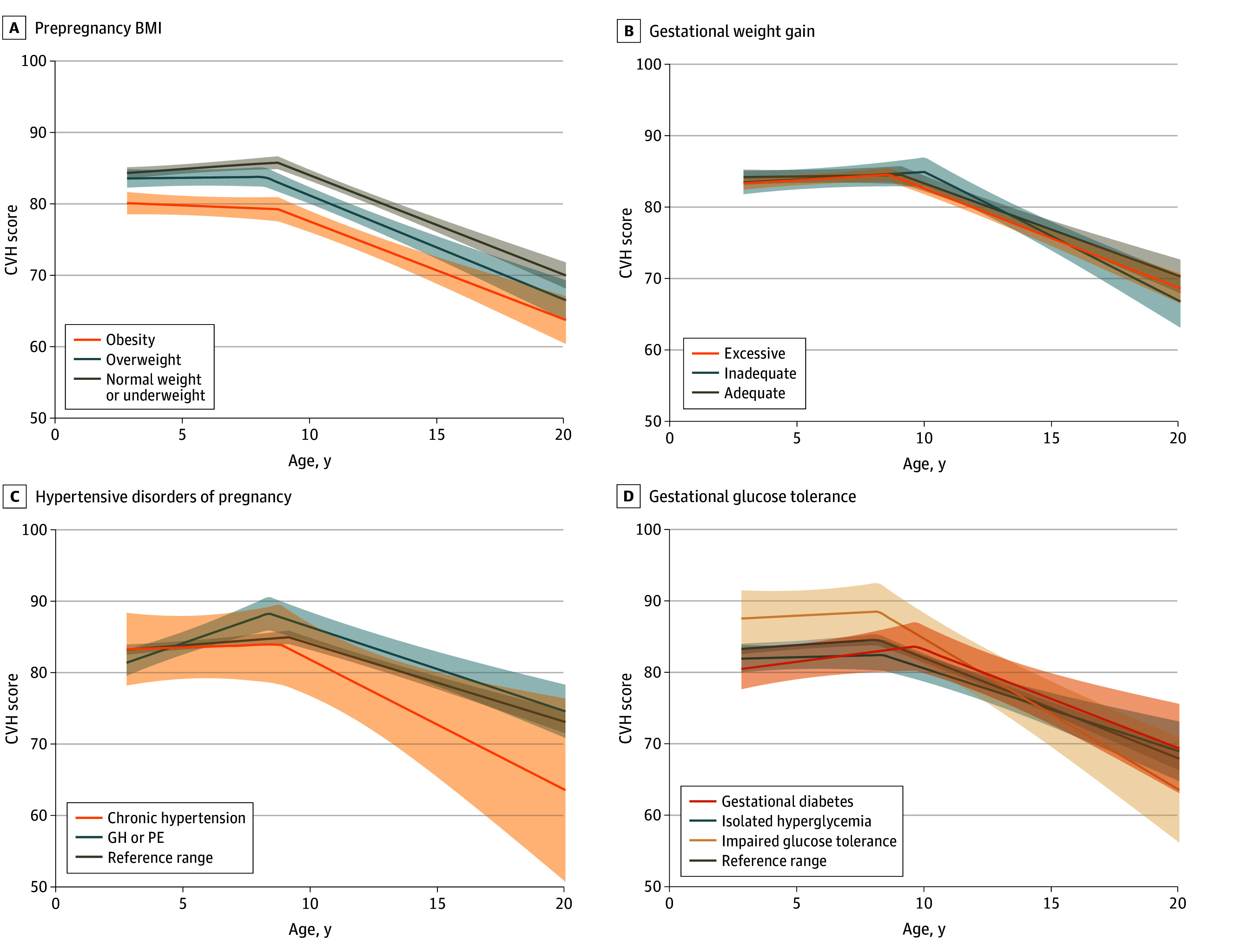
Unadjusted Trajectories of Overall Cardiovascular Health (CVH) Scores From Early Childhood to Late Adolescence Trajectories according to prepregnancy body mass index (A), gestational weight gain (B), hypertensive disorders of pregnancy (C), and gestational glucose tolerance (D). Shaded areas indicate 95% CIs. BMI indicates body mass index (calculated as weight in kilograms divided by height in meters squared); GH, gestational hypertension; PE, preeclampsia.

After adjusting for covariates, prepregnancy overweight or obesity (vs healthy or underweight) was associated with lower CVH from childhood to adolescence (Figure 1); the mean CVH score difference for children born to mothers with prepregnancy obesity (vs healthy or underweight) was −1.3 (95% CI, −2.0 to −0.6) at 3 years, −1.9 (95% CI,−2.8 to −1.0) at 8 years, −2.0 (95% CI,−2.9 to −1.2) at 13 years, and −0.9 (95% CI,−1.4 to −0.4) at 18 years (Table 1). Prepregnancy obesity (vs healthy or underweight) was also associated with later timing of inflection (β = 0.1; 95% CI. 0.0 to 0.2 years) and slower CVH decline after inflection (0.2; 95% CI, 0.1 to 0.4 points per year). Excessive (vs adequate) GWG was associated with lower CVH score at 3 years only (−0.6; 95% CI,−1.1 to −0.1) but was not associated with any CVH trajectory parameter (Table 2).

**Table 1.** Association of Prenatal and Perinatal Factors With Projected Overall Cardiovascular Health Scores at Age 3, 8, 13, and 18 Years (n = 1338)

Factor	β (95% CI)^a^			
	3 y	8 y	13 y	18 y
Prepregnancy body mass index^b^^,^^c^				
Healthy plus underweight	[Reference]	[Reference]	[Reference]	[Reference]
Overweight	−0.6 (−1.2 to 0.0)	−0.8 (−1.5 to −0.1)^d^	−0.8 (−1.5 to −0.2)^d^	−0.3 (−0.7 to 0.1)
Obesity	−1.3 (−2.0 to −0.6)^d^	−1.9 (−2.8 to −1.0)^d^	−2.0 (−2.9 to −1.2)^d^	−0.9 (−1.4 to −0.4)^d^
Gestational weight gain^b^				
Adequate	[Reference]	[Reference]	[Reference]	[Reference]
Inadequate	−0.4 (−1.2 to 0.4)	0.2 (−0.8 to 1.2)	0.3 (−0.7 to 1.2)	−0.2 (−0.8 to 0.4)
Excessive	−0.6 (−1.1 to −0.1)^d^	−0.0 (−0.7 to 0.6)	0.0 (−0.6 to 0.7)	−0.3 (−0.6 to 0.1)
Hypertenstive disorders of pregnancy^e^				
Normal blood pressure	[Reference]	[Reference]	[Reference]	[Reference]
Gestational hypertension or preeclampsia	0.0 (−0.8 to 0.8)	1.4 (0.4 to 2.3)	1.4 (0.5 to 2.3)	0.1 (−0.5 to 0.6)
Chronic hypertension	−0.4 (−2.4 to 1.6)	−0.4 (−2.8 to 2.0)	0.8 (−1.5 to 3.0)	1.1 (−0.3 to 2.5)
Gestational glucose tolerance^e^				
Normal glucose tolerance	[Reference]	[Reference]	[Reference]	[Reference]
Isolated hyperglycemia	−0.3 (−1.2 to 0.5)	−1.2 (−2.3 to −0.1)^d^	−1.1 (−2.1 to −0.1)^d^	−0.4 (−1.0 to 0.2)
Impaired glucose tolerance	1.0 (−0.3 to 2.4)	2.1 (0.5 to 3.8)	1.3 (−0.2 to 2.9)	0.2 (−0.8 to 1.1)
Gestational diabetes	−0.7 (−1.8 to 0.4)	0.3 (−1.1 to 1.6)	0.9 (−0.4 to 2.1)	0.1 (−0.7 to 0.9)
Prenatal smoking^f^				
Never	[Reference]	[Reference]	[Reference]	[Reference]
Before pregnancy	−0.1 (−0.8 to 0.5)	−0.9 (−1.6 to −0.1)^d^	−0.6 (−1.4 to 0.1)	0.0 (−0.4 to 0.4)
During pregnancy	−1.4 (−2.3 to −0.6)^d^	−1.7 (−2.7 to −0.7)^d^	−1.4 (−2.4 to −0.5)^d^	−0.8 (−1.3 to −0.2)^d^
Breastfeeding initiation^g^				
Yes	[Reference]	[Reference]	[Reference]	[Reference]
No	−0.6 (−1.4 to 0.2)	−0.5 (−1.5 to 0.5)	−0.4 (−1.3 to 0.6)	−0.2 (−0.8 to 0.4)
Infant feeding type in the first 6 mo^g^				
Breastfeeding	[Reference]	[Reference]	[Reference]	[Reference]
Weaned	−0.7 (−1.3 to 0.0)^d^	−1.0 (−1.8 to −0.3)^d^	−0.9 (−1.6 to −0.2)^d^	−0.4 (−0.8 to 0.1)
Mixed	−0.5 (−1.1 to 0.2)	−0.3 (−1.1 to 0.5)	−0.3 (−1.1 to 0.5)	−0.2 (−0.7 to 0.3)
Formula only	−1.1 (−2.0 to −0.2)^d^	−1.2 (−2.2 to −0.1)^d^	−0.9 (−1.9 to 0.2)	−0.4 (−1.0 to 0.2)

^a^ All β estimates represent the mean difference in CVH score at age 3, 8, 13, or 18 years when comparing 1 category of the prenatal or perinatal factor with its corresponding reference category.

^b^ Adjusted for maternal age at enrollment, parity, race and ethnicity, education level, household income, and prenatal smoking.

^c^ Body mass index is calculated as weight in kilograms divided by height in meters squared.

^d^ *P* < .05.

^e^ Adjusted for maternal age at enrollment, parity, race and ethnicity, education level, household income, prenatal smoking, and prepregnancy body mass index.

^f^ Adjusted for maternal age at enrollment, parity, race and ethnicity, education level, and household income.

^g^ Adjusted for maternal age at enrollment, parity, race and ethnicity, education level, household income, prenatal smoking, prepregnancy body mass index, gestational age at delivery, and birth weight-for-gestational-age *z* score.

**Table 2.** Association of Prenatal and Perinatal Factors With Overall Cardiovascular Health (CVH) Trajectory Parameters (n = 1338)

Factor	β (95% CI)^a^		
	Slope of CVH before inflection, points/y	Timing of inflection, point, y	Slope of CVH after inflection, points/y
Prepregnancy body mass index^b^^,^^c^			
Healthy plus underweight	[Reference]	[Reference]	[Reference]
Overweight	−0.1 (−0.2 to 0.1)	0.0 (0.0 to 0.1)	0.1 (0.0 to 0.3)^d^
Obesity	−0.1 (−0.3 to 0.1)	0.1 (0.0 to 0.2)^d^	0.2 (0.1 to 0.4)^d^
Gestational weight gain^b^			
Adequate	[Reference]	[Reference]	[Reference]
Inadequate	0.1 (−0.1 to 0.3)	0.0 (−0.1 to 0.1)	−0.1 (−0.3 to 0.1)
Excessive	0.1 (−0.0 to 0.3)	0.0 (−0.1 to 0.1)	−0.1 (−0.2 to 0.1)
Hypertenstive disorders of pregnancy^e^			
Normal blood pressure	[Reference]	[Reference]	[Reference]
Gestational hypertension or preeclampsia	0.3 (0.1 to 0.5)^d^	−0.1 (−0.2 to 0.0)^d^	−0.3 (−0.5 to −0.1)^d^
Chronic hypertension	−0.0 (−0.5 to 0.5)	−0.1 (−0.4 to 0.2)	0.1 (−0.4 to 0.5)
Gestational glucose tolerance^e^			
Normal glucose tolerance	[Reference]	[Reference]	[Reference]
Isolated hyperglycemia	−0.1 (−0.4 to 0.1)	0.1 (0.0 to 0.2)^d^	0.1 (−0.1 to 0.4)
Impaired glucose tolerance	0.2 (−0.2 to 0.6)	−0.2 (−0.4 to 0.0)^d^	−0.2 (−0.6 to 0.1)
Gestational diabetes	0.2 (−0.1 to 0.5)	0.1 (−0.1 to 0.2)	−0.1 (−0.4 to 0.1)
Prenatal smoking^f^			
Never	[Reference]	[Reference]	[Reference]
Before pregnancy	−0.1 (−0.3 to 0.0)	0.1 (0.0 to 0.2)^d^	0.1 (−0.0 to 0.3)
During pregnancy	−0.0 (−0.3 to 0.2)	0.2 (0.1 to 0.3)^d^	0.1 (−0.1 to 0.3)
Breastfeeding initiation^g^			
Yes	[Reference]	[Reference]	[Reference]
No	0.0 (−0.2 to 0.2)	0.0 (−0.1 to 0.1)	0.0 (−0.2 to 0.2)
Infant feeding type in the first 6 mo^g^			
Breastfeeding	[Reference]	[Reference]	[Reference]
Weaned	−0.1 (−0.2 to 0.1)	0.1 (0.0 to 0.2)	0.1 (−0.1 to 0.3)
Mixed	0.0 (−0.2 to 0.2)	0.0 (−0.1 to 0.1)	0.0 (−0.1 to 0.2)
Formula only	0.0 (−0.2 to 0.2)	0.1 (−0.1 to 0.2)	0.1 (−0.1 to 0.3)

^a^ All β estimates represent the mean difference in CVH parameters (ie, slope of CVH before inflection, timing of inflection point, or slope of CVH after inflection) when comparing 1 category of the prenatal or perinatal factor with its corresponding reference category.

^b^ Adjusted for maternal age at enrollment, parity, race and ethnicity, education level, household income, and prenatal smoking.

^c^ Body mass index is calculated as weight in kilograms divided by height in meters squared.

^d^ *P* < .05.

^e^ Adjusted for maternal age at enrollment, parity, race and ethnicity, education level, household income, prenatal smoking, and prepregnancy body mass index.

^f^ Adjusted for maternal age at enrollment, parity, race and ethnicity, education level, and household income.

^g^ Adjusted for maternal age at enrollment, parity, race and ethnicity, education level, household income, prenatal smoking, prepregnancy body mass index, gestational age at delivery, and birth weight-for-gestational-age *z* score.

**Figure 2.**
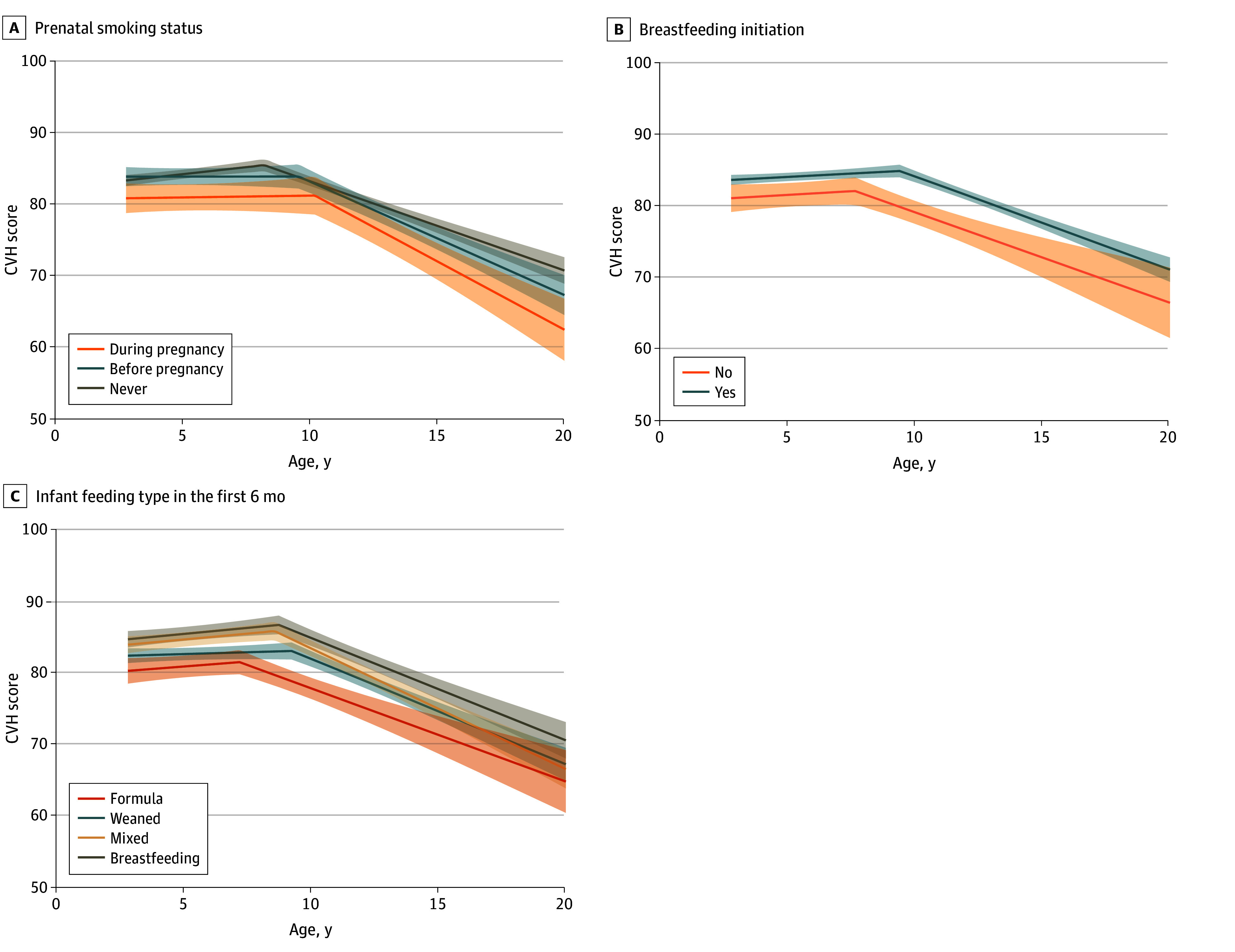
Unadjusted Trajectories of Overall Cardiovascular Health (CVH) Score From Early Childhood to Late Adolescence Trajectories according to prenatal smoking status (A), breastfeeding initiation (B), and infant feeding type in the first 6 months (C). Shaded areas indicate 95% CIs.

Gestational hypertension or preeclampsia (vs normal blood pressure) was associated with faster CVH gain before inflection (β = 0.3; 95% CI, 0.1 to 0.5 points per year), earlier timing of inflection (−0.1; 95% CI, −0.2 to 0.0 years), and faster CVH decline after inflection (−0.3; 95% CI, −0.5 to −0.1 points per year). Isolated hyperglycemia (vs normal glucose tolerance) was associated with lower CVH at 8 years and 13 years as well as later timing of inflection, while impaired glucose tolerance was associated with earlier timing of inflection only (Table 1 and Table 2). Children born to mothers who smoked during pregnancy (vs never) exhibited lower CVH from childhood to adolescence (Figure 2) and later timing of inflection (β = 0.2; 95% CI, 0.1 to 0.3 years) (Table 2). Children who were weaned or formula-fed only (vs breastfeeding) in the first 6 months also had lower CVH from childhood to adolescence (Figure 2 and Table 1) but showed no associations with CVH trajectory parameters (Table 2).

### Behavioral CVH

The decline in overall CVH appeared to be attributed primarily to behavioral CVH (eFigures 3-4 in Supplement 1). Children who were weaned or formula-fed only (vs breastfeeding) in the first 6 months exhibited lower behavioral CVH from childhood to adolescence (eTable 4 in Supplement 1). Those who were weaned (vs breastfeeding) in the first 6 months had later timing of inflection, while those who were formula-fed only (vs breastfeeding) in the first 6 months exhibited slower behavioral CVH gain before inflection and later timing of inflection (eTable 5 in Supplement 1). Other factors were not associated with behavioral CVH.

### Biological CVH

Prepregnancy overweight or obesity (vs healthy or underweight) and smoking during pregnancy (vs never) were each associated with lower biological CVH from childhood to adolescence (eFigures 5-6 in Supplement 1) and slower biological CVH decline after inflection (eTable 7 in Supplement 1). Excessive (vs adequate) GWG was associated with lower biological CVH from childhood to adolescence, while isolated hyperglycemia (vs normal glucose tolerance) was associated with lower biological CVH during childhood only and slower biological CVH decline after inflection (eTables 6-7 in Supplement 1). Gestational hypertension or preeclampsia (vs normal blood pressure) was associated with faster CVH decline after inflection (eTable 7 in Supplement 1). Results from the sensitivity analyses were largely similar to the main analyses, albeit with some differences observed in the associations of prepregnancy obesity with biological CVH trajectory parameters (eTables 8-13, eFigures 7-8, and eResults in Supplement 1).

## Discussion

In this cohort study, we found that prepregnancy overweight or obesity, smoking during pregnancy, and formula-feeding in the first 6 months of life were each associated with lower overall CVH from childhood to adolescence. We also identified significant differences—albeit small in magnitude—in overall CVH trajectory parameters (eg, timing and rate of CVH decline) according to prepregnancy BMI, pregnancy conditions, and prenatal smoking status.

Our results corroborate previous findings from other studies. In Denver, 2 studies showed that prepregnancy overweight or obesity was associated with lower overall CVH and lower odds of ideal CVH in children aged 4 to 7 years.^21,22^ In the Hyperglycemia and Adverse Pregnancy Outcome (HAPO) study, Perak et al^23^ reported that nonideal maternal BMI at approximately 28 weeks of gestation (defined as BMI >28.5) and prenatal smoking were each associated with lower odds of ideal CVH in adolescents aged 10 to 14 years. Furthermore, we noted that GD was not associated with CVH in childhood or adolescence, consistent with findings from the HAPO study,^23^ although children of women with isolated hyperglycemia during pregnancy had lower CVH in midchildhood and early adolescence. These observations could be explained by the fact that women diagnosed with GD in Project Viva received education and/or treatment with diet and exercise during pregnancy, while women with isolated hyperglycemia were not managed or treated.^32^

Our study has important distinctions from others that are worth noting. Prior studies examined offspring CVH either in childhood^21,22^ or adolescence^23^ only which did not allow for a detailed assessment of how the associations with CVH may change over time. Additionally, 2 studies^22,23^ examined child CVH using the Life’s Simple 7 (LS7) construct, which compared with LE8 is less sensitive to interindividual and intraindividual differences in CVH^3^ that may be important during early life. Furthermore, the HAPO study examined associations with CVH using the LS7 biological factors only,^23^ primarily because the LS7 behavioral factors do not lend themselves to fully continuous scales.^3,4^ Our study directly addressed these research gaps by (1) examining CVH repeatedly from early childhood to late adolescence, enabling us to characterize associations with CVH over time; and (2) using the LE8 construct, which allowed us to determine the extent to which associations with overall CVH may be associated with behavioral or biological CVH. Altogether, our study contributes novel evidence to the extant literature linking prenatal and perinatal risk factors with subsequent CVH in children.

We observed that certain risk factors were associated with CVH trajectory parameters in the opposite direction than hypothesized (eg, prepregnancy obesity and prenatal smoking were associated with later timing and slower rate of decline in overall CVH). It is difficult to compare and interpret these findings in the context of current literature, as no studies to our knowledge have characterized associations between prenatal and perinatal risk factors and CVH trajectories in children or between CVH inflection point or slope parameters and subsequent health outcomes. Although we have no clear biological explanation for our findings, the magnitude of associations with CVH trajectory parameters were quite small (ie, 0.1-0.2 years later timing of inflection and 0.1-0.3 points per year slower decline in CVH). In line with our initial hypothesis, prepregnancy obesity, smoking during pregnancy, and formula feeding in the first 6 months were each associated with lower overall CVH by 1 to 2 points from childhood to adolescence. A recent study^11^ reported that a 1-point lower CVH in adolescence was associated with approximately 20% higher incidence in CVD and mortality. Thus, while the clinical implications of small differences in CVH inflection point or slope parameters remain unclear and merit further research, the sustained CVH score differences observed in our study may have substantial long-term health consequences.

Consistent with our previous work,^6^ we observed that the overall and behavioral CVH trajectories demonstrated similar patterns of CVH decline beginning at approximately 10 years of age across categories of prenatal and perinatal factors. This finding suggests that the decline in overall CVH in children is largely associated with behavioral CVH, which likely reflects emerging autonomy from parental influence^33^ and developmental transitions occurring during these life stages that may result in declining health behaviors (eg, adoption of risk-taking behaviors such as smoking or vaping),^34,35^ or changes in school schedules that may interfere with meeting guidelines for healthy sleep duration, physical activity, and/or diet.^36^

Our results highlight the potential different pathways in which prenatal and perinatal risk factors may be associated with child CVH over time. We observed that the association of prepregnancy overweight or obesity and prenatal smoking with lower overall CVH from childhood to adolescence was attributed mainly to lower biological CVH. Specifically, these findings may be associated with higher BMI and BP; in Project Viva, we have previously shown that higher prepregnancy BMI and prenatal smoking were each associated with higher BMI and BP trajectories from childhood to adolescence^13,14^ but were not associated with other biological CVH factors including glucose and cholesterol.^37^ We also noted that being weaned or formula-fed only in the first 6 months was associated with lower overall CVH from childhood to adolescence, which was mainly associated with lower behavioral CVH. These results may be specifically attributed to poorer diet and shorter sleep duration; in Project Viva, we have shown that early weaning or no breastfeeding in infancy were each associated with lower diet quality scores^38^ and shorter sleep duration.^39^ Beyond these individual CVH metrics, it is also possible that unfavorable social (eg, family dysfunction,^40^ chronic stress,^41^ and/or mood disorders^42^) and structural (eg, residence in disadvantaged neighborhoods,^43^ lack of access to healthy foods^44^ or greenspace^45,46^) factors that have been linked with CVD precursors and shared between the birthing person and their offspring may underlie our observed associations between prenatal and perinatal risk factors and offspring CVH. Future studies in other settings could be done to explore these relationships.

### Strengths and Limitations

Strengths of our study include its prospective study design, almost 2 decades of follow-up, objective CVH measures obtained by highly trained staff using standardized protocols, and use of innovative statistical methods to characterize CVH trajectories. This study, however, has its limitations. We only had data on 6 CVH metrics instead of 8 in early childhood, and some children had missing CVH scores at certain life stages. However, we do not anticipate these issues to substantially affect estimation of CVH trajectories because segmented mixed models allow for estimation of trajectory parameters in the presence of missing data via maximum likelihood estimation.^29,30^ We excluded 37% of children from the original cohort due to missing data, and they were more likely to be from families of lower socioeconomic status; our findings thus may not be generalizable to these children. Our findings may also not be generalizable to populations from different settings, because most participants lived in eastern Massachusetts, had health care coverage, and had higher CVH relative to the general US child population.^4^

## Conclusions

Our findings provide evidence of associations of known prenatal and perinatal risk factors of childhood CVD precursors, including prepregnancy obesity, smoking during pregnancy, and formula-feeding in the first 6 months of life, with adverse CVH trajectories from early childhood to late adolescence. Importantly, these factors are amenable to behavior-change interventions. Given that higher CVH from childhood onwards confers long-term health benefits,^7,8,9,10,11,12^ future studies are warranted to examine whether intervening on these factors would be effective in changing CVH in children, and if so, whether such interventions can avert the loss of and preserve CVH across childhood and adolescence.

## Notes

Supplement 1. **eMethods. **Assessment of CVH metrics, quantification of cardiovascular health (CVH) score, and characterizing CVH trajectories using segmented mixed-effect models. **eResults.** Sensitivity analyses for overall, behavioral, and biological CVH. **eTable 1.** Life’s Essential 8 scoring algorithm for calculating cardiovascular health (CVH) scores for each CVH metric from early childhood to late adolescence. **eTable 2. **Participant characteristics. **eTable 3. **Trajectory parameters for overall, behavioral, and biological CVH in males and females (n=1,333). **eTable 4. **Association of prenatal and perinatal factors with projected behavioral cardiovascular health (CVH) scores at 3, 8, 13, and 18 years (n=1,310). **eTable 5.** Association of prenatal and perinatal factors with behavioral cardiovascular health (CVH) trajectory parameters (n=1,310). **eTable 6.** Association of prenatal and perinatal factors with projected biological cardiovascular health (CVH) scores at 3, 8, 13, and 18 years (n=1,286). **eTable 7.** Association of prenatal and perinatal factors with biological cardiovascular health (CVH) trajectory parameters (n=1,286). **eTable 8.** Sensitivity analyses for the association of prenatal and perinatal factors with projected overall cardiovascular health (CVH) scores at 3, 8, 13, and 18 year in a subset of children with all available CVH metrics at each life stage (n=1,079). **eTable 9.** Sensitivity analyses for the association of prenatal and perinatal factors with overall cardiovascular health (CVH) trajectory parameters in a subset of children with all available CVH metrics at each life stage (n=1,079). **eTable 10.** Sensitivity analyses for the association of prenatal and perinatal factors with projected behavioral cardiovascular health (CVH) scores at 3, 8, 13, and 18 years in a subset of children with all available CVH metrics at each life stage (n=1,079). **eTable 11.** Sensitivity analyses for the association of prenatal and perinatal factors with behavioral cardiovascular health (CVH) trajectory parameters in a subset of children with all available CVH metrics at each life stage (n=1,079). **eTable 12.** Sensitivity analyses for the association of prenatal and perinatal factors with projected biological cardiovascular health (CVH) scores at 3, 8, 13, and 18 years in a subset of children with all available CVH metrics at each life stage (n=1,079) **eTable 13.** Sensitivity analyses for the association of prenatal and perinatal factors with biological cardiovascular health (CVH) trajectory parameters in a subset of children with all available CVH metrics at each life stage (n=1,079) **eFigure 1.** Flowchart for analytic sample **eFigure 2.** Directed acyclic graph describing the relationship of prenatal and perinatal factors, covariates, and cardiovascular health in children **eFigure 3.** Unadjusted trajectories of behavioral cardiovascular health (CVH) scores from early childhood to late adolescence according to prepregnancy body mass index (A), gestational weight gain (B), hypertensive disorders of pregnancy (C), and gestational glucose tolerance (D) **eFigure 4.** Unadjusted trajectories of behavioral cardiovascular health (CVH) score from early childhood to late adolescence according to prenatal smoking status (A), breastfeeding initiation (B), and infant feeding type in the first 6 months (C) **eFigure 5.** Unadjusted trajectories of biological cardiovascular health (CVH) scores from early childhood to late adolescence according to prepregnancy body mass index (A), gestational weight gain (B), hypertensive disorders of pregnancy (C), and gestational glucose tolerance (D) **eFigure 6.** Unadjusted trajectories of biological cardiovascular health (CVH) score from early childhood to late adolescence according to prenatal smoking status (A), breastfeeding initiation (B), and infant feeding type in the first 6 months (C) **eFigure 7. **Unadjusted trajectories of overall cardiovascular health (CVH) scores from early childhood to late adolescence according to prepregnancy body mass index (A), gestational weight gain (B), hypertensive disorders of pregnancy (C), and gestational glucose tolerance (D) **eFigure 8.** Unadjusted trajectories of overall cardiovascular health (CVH) scores from early childhood to late adolescence according to prenatal smoking status (A), breastfeeding initiation (B), and infant feeding type in the first 6 months (C). Trajectories are restricted to a subset of children (n=1,079) with all available CVH metrics at each life stage

Supplement 2.
Data Sharing Statement
