# Supplementary material for: Prenatal and Perinatal Factors of Life’s Essential 8 Cardiovascular Health Trajectories
Source: JAMA Netw Open. 2025 Apr 29;8(4):e257774. doi: 10.1001/jamanetworkopen.2025.7774 (PMC12042050; doi:10.1001/jamanetworkopen.2025.7774)
Supplement: Supplement 1. — eMethods. Assessment of CVH metrics, quantification of cardiovascular health (CVH) score, and characterizing CVH trajectories using segmented mixed-effect models. eResults. Sensitivity analyses for overall, behavioral, and biological CVH. eTable 1. Life’s Essential 8 scoring algorithm for calculating cardiovascular health (CVH) scores for each CVH metric from early childhood to late adolescence. eTable 2. Participant characteristics. eTable 3. Trajectory parameters for overall, behavioral, and biological CVH in males and females (n=1,333). eTable 4. Association of prenatal and perinatal factors with projected behavioral cardiovascular health (CVH) scores at 3, 8, 13, and 18 years (n=1,310). eTable 5. Association of prenatal and perinatal factors with behavioral cardiovascular health (CVH) trajectory parameters (n=1,310). eTable 6. Association of prenatal and perinatal factors with projected biological cardiovascular health (CVH) scores at 3, 8, 13, and 18 years (n=1,286). eTable 7. Association of prenatal and perinatal factors with biological cardiovascular health (CVH) trajectory parameters (n=1,286). eTable 8. Sensitivity analyses for the association of prenatal and perinatal factors with projected overall cardiovascular health (CVH) scores at 3, 8, 13, and 18 year in a subset of children with all available CVH metrics at each life stage (n=1,079). eTable 9. Sensitivity analyses for the association of prenatal and perinatal factors with overall cardiovascular health (CVH) trajectory parameters in a subset of children with all available CVH metrics at each life stage (n=1,079). eTable 10. Sensitivity analyses for the association of prenatal and perinatal factors with projected behavioral cardiovascular health (CVH) scores at 3, 8, 13, and 18 years in a subset of children with all available CVH metrics at each life stage (n=1,079). eTable 11. Sensitivity analyses for the association of prenatal and perinatal factors with behavioral cardiovascular health (CVH) trajec [file jamanetwopen-e257774-s001.pdf]

Supplemental Online Content

Aris IM, Rifas-Shiman SL, de Ferranti SD, Hivert M-F, Perng W. Pre- and perinatal factors of Life's Essential 8 cardiovascular health trajectories. *JAMA Netw Open*. 2025;8(4):e257774. doi:10.1001/jamanetworkopen.2025.7774

eMethods. Assessment of CVH metrics, quantification of cardiovascular health (CVH) score, and characterizing CVH trajectories using segmented mixed-effect models.

eResults. Sensitivity analyses for overall, behavioral, and biological CVH.

eTable 1. Life's Essential 8 scoring algorithm for calculating cardiovascular health (CVH) scores for each CVH metric from early childhood to late adolescence.

eTable 2. Participant characteristics.

eTable 3. Trajectory parameters for overall, behavioral, and biological CVH in males and females (n=1,333).

eTable 4. Association of pre- and perinatal factors with projected behavioral cardiovascular health (CVH) scores at 3, 8, 13, and 18 years (n=1,310).

eTable 5. Association of pre- and perinatal factors with behavioral cardiovascular health (CVH) trajectory parameters (n=1,310).

eTable 6. Association of pre- and perinatal factors with projected biological cardiovascular health (CVH) scores at 3, 8, 13, and 18 years (n=1,286).

eTable 7. Association of pre- and perinatal factors with biological cardiovascular health (CVH) trajectory parameters (n=1,286).

eTable 8. Sensitivity analyses for the association of pre- and perinatal factors with projected overall cardiovascular health (CVH) scores at 3, 8, 13, and 18 year in a subset of children with all available CVH metrics at each life stage (n=1,079).

eTable 9. Sensitivity analyses for the association of pre- and perinatal factors with overall cardiovascular health (CVH) trajectory parameters in a subset of children with all available CVH metrics at each life stage (n=1,079).

eTable 10. Sensitivity analyses for the association of pre- and perinatal factors with projected behavioral cardiovascular health (CVH) scores at 3, 8, 13, and 18 years in a subset of children with all available CVH metrics at each life stage (n=1,079).

eTable 11. Sensitivity analyses for the association of pre- and perinatal factors with behavioral cardiovascular health (CVH) trajectory parameters in a subset of children with all available CVH metrics at each life stage (n=1,079).

© 2025 Aris IM et al. *JAMA Network Open*.

eTable 12. Sensitivity analyses for the association of pre- and perinatal factors with projected biological cardiovascular health (CVH) scores at 3, 8, 13, and 18 years in a subset of children with all available CVH metrics at each life stage (n=1,079)

eTable 13. Sensitivity analyses for the association of pre- and perinatal factors with biological cardiovascular health (CVH) trajectory parameters in a subset of children with all available CVH metrics at each life stage (n=1,079)

eFigure 1. Flowchart for analytic sample

eFigure 2. Directed acyclic graph describing the relationship of pre- and perinatal factors, covariates, and cardiovascular health in children

eFigure 3. Unadjusted trajectories of behavioral cardiovascular health (CVH) scores from early childhood to late adolescence according to prepregnancy body mass index (A), gestational weight gain (B), hypertensive disorders of pregnancy (C), and gestational glucose tolerance (D)

eFigure 4. Unadjusted trajectories of behavioral cardiovascular health (CVH) score from early childhood to late adolescence according to prenatal smoking status (A), breastfeeding initiation (B), and infant feeding type in the first 6 months (C)

eFigure 5. Unadjusted trajectories of biological cardiovascular health (CVH) scores from early childhood to late adolescence according to pre-pregnancy body mass index (A), gestational weight gain (B), hypertensive disorders of pregnancy (C), and gestational glucose tolerance (D)

eFigure 6. Unadjusted trajectories of biological cardiovascular health (CVH) score from early childhood to late adolescence according to prenatal smoking status (A), breastfeeding initiation (B), and infant feeding type in the first 6 months (C)

eFigure 7. Unadjusted trajectories of overall cardiovascular health (CVH) scores from early childhood to late adolescence according to pre-pregnancy body mass index (A), gestational weight gain (B), hypertensive disorders of pregnancy (C), and gestational glucose tolerance (D)

eFigure 8. Unadjusted trajectories of overall cardiovascular health (CVH) scores from early childhood to late adolescence according to prenatal smoking status (A), breastfeeding initiation (B), and infant feeding type in the first 6 months (C). Trajectories are restricted to a subset of children (n=1,079) with all available CVH metrics at each life stage

This supplemental material has been provided by the authors to give readers additional information about their work.

© 2025 Aris IM et al. *JAMA Network Open*.

eMethods

Assessment of CVH metrics

Behavioral factors

Trained research assistants collected information on dietary intake using a validated 84-item food frequency questionnaire (FFQ) in early childhood, PrimeScreen (an 18-item validated FFQ) in mid-childhood and early adolescence, and the Automated Self-Administered 24-hour (ASA-24) Dietary Recall in late adolescence. We extracted information on food intake for FFQs using the Harvard nutrient composition database, and nutrient and caloric intake from the ASA database for ASA-24. We used this information to calculate the Healthy-Eating-Index-2015 score across all life stages.

The AHA's nicotine exposure metric is for children aged 12–19y; hence, we used exposure to indoor smoking as a CVH metric for children <12y. In early and mid-childhood, mothers reported their child's exposure to indoor tobacco smoke using questionnaires. In early and late adolescence, children self-reported whether they have ever smoked using interviewer-administered questionnaires.

Using validated questionnaires, mothers reported the amount of time their children engaged in active play, recreational activities, or physical activity in the past month from early childhood to early adolescence, and children self-reported this information in late adolescence, which we used to calculate hours/day of light-to-moderate and vigorous physical activity. Mothers also reported their child's sleep duration in a usual 24-hour period from early childhood to early adolescence, and children also self-reported this information in late adolescence using validated questionnaires.

Biological factors

From early childhood to late adolescence, trained research assistants measured child's weight on a calibrated electronic scale and height via a stadiometer and recorded systolic and diastolic BP on the child's upper arm up to 5 times at 1-minute intervals using biannually calibrated oscillometric automated monitors [in childhood: Dinamap Pro-100 (Dinamap); in adolescence: Omron HEM-907XL (Omron, Bannockburn, Illinois)] following standardized procedures. We calculated BMI and derived age- and sex-specific BMI percentiles using the Centers for Disease Control and Prevention growth reference. We averaged the 5 BP measurements at each visit and derived age-, sex-, and height-specific BP percentiles using the 2017 US child BP reference.

Trained technicians collected fasting blood specimens from mid-childhood to late adolescence. We measured fasting glucose enzymatically (Roche Diagnostics, Indianapolis, IN), and assessed plasma total and high-density lipoprotein (HDL) cholesterol using an enzymatic method with correction for endogenous glycerol. We calculated non-HDL cholesterol as total cholesterol – HDL cholesterol.

Quantification of cardiovascular health (CVH) score

We measured up to 6 CVH metrics (diet, nicotine exposure, physical activity, sleep duration, BMI, and BP) in early childhood, and up to 8 metrics (diet, nicotine exposure, physical activity, sleep duration, BMI, BP, fasting glucose, and non-HDL cholesterol) from mid-childhood to late adolescence. We calculated the overall CVH score by summing the scores for all metrics available at each life stage, and dividing by the denominator of the number of metrics available at each life stage. For example, among children with data on all 6 CVH metrics in early

© 2025 Aris IM et al. *JAMA Network Open*.

childhood, we calculated the overall CVH score by summing the scores for the 6 CVH metrics and dividing the total by 6, to provide a LE8 score ranging from 0 to 100. Among those with data on only 3 CVH metrics in early childhood, we calculated the overall CVH score by summing the scores for the 3 CVH metrics and dividing the total by 3. We also calculated CVH scores for behavioral and biological CVH factors separately at each life stage. For example, in early childhood, we measured 4 behavioral and 2 biological factors. Thus, among children with data on all 6 CVH metrics in early childhood, we calculated the behavioral CVH score by summing the scores for the 4 behavioral metrics and dividing the total by 4, and calculated the biological CVH score by summing the scores for the 2 behavioral metrics and dividing the total by 2. We used the same approach to calculate overall, behavioral, and biological CVH scores at other life stages.

Characterizing cardiovascular health (CVH) trajectories using segmented mixed-effect models

Segmented mixed models extend simple segmented (i.e., piecewise) regression to the mixed-effects model framework by including random effects in each model parameter. Importantly, segmented mixed models enable estimation of trajectory parameters even in the presence of missing data via maximum likelihood estimation. This method enables estimation of CVH trajectories even if CVH scores are available at only one timepoint, with the caveat that children with missing CVH scores at multiple life stages would have trajectories closer to the population average. The model is given by the formula:

$$y_{ij} = \beta_{0i} + \beta_{1i}(t_{ij}) + \delta_i(t_{ij} - \psi_i)_+ + \varepsilon_{ij},$$

where y_{ij} is the repeated CVH score measure over time for subject i, t_{ij} is the age when y_{ij} occurs, and $(t_{ij} - \psi_i)_+$ represents the linear spline function $(t - \psi)I(t > \psi)$, where $I(t > \psi)$ is an indicator argument equaling one when $t > \psi$ and zero otherwise. For each subject i, the parameter β_{0i} is the mean CVH score at time $t = 0$, ψ_i is the timing of the inflection point when CVH trajectory begins to accelerate or decelerate; β_{1i} is the linear slope for CVH trajectory when $t \leq \psi$ (i.e., the slope of CVH before the inflection point); and δ_i is the difference-in-slopes. The sum of β_{1i} and δ_i is equivalent to the linear slope for CVH trajectory when $t > \psi$ (i.e., the slope of CVH after the inflection point).

To characterize sex-specific trajectories of CVH score from early childhood to late adolescence, we first fitted a linear mixed-effect model for overall CVH score with child sex, child age, and an interaction term between the two variables as fixed effects, as well as random effects for the intercept and slope using the nlme package in R. Subsequently, we updated the initial mixed-effect model with the segmented.lme package in R, which implements an iterative procedure to estimate the fixed and random effects for the inflection point (ψ) and the difference-in-slopes (δ) parameters of the CVH trajectory using a maximum likelihood approach. We determined the best fitting model using Bayesian Information Criteria and likelihood ratio tests. Finally, we used the fitted model to derive subject-specific estimates of the slope of CVH trajectory before the inflection point (β_{1i}), timing of the inflection point when CVH trajectory begins to accelerate or decelerate (ψ_i), and slope of CVH trajectory after the inflection point ($\beta_{1i} + \delta_i$) by calculating the sum of the fixed and random effects for each of these trajectory parameters. We also used these models to estimate the projected CVH score over time for each child, and subsequently plotted the average trajectory of CVH score for each pre- and perinatal factor. Further, as we did not observe significant interactions of each pre- and perinatal risk factor with child sex, we plotted non-sex-specific CVH trajectory patterns for each pre- and perinatal factor.

© 2025 Aris IM et al. *JAMA Network Open*.

eResults

Sensitivity analyses for overall CVH

Results restricted to children with all available CVH metrics at any life stage did not differ from our main analyses. Specifically, pre-pregnancy overweight or obesity (vs. healthy or underweight), smoking during pregnancy (vs. never), and being weaned or formula-fed only in the first 6 months (vs. breastfeeding) were each associated with lower CVH from childhood to adolescence (**eFigures 7–8** and **eTable 8**). Similarly, pre-pregnancy obesity (vs. healthy or underweight) was associated with later timing of inflection, while IGT (vs. normal glucose tolerance) was associated with earlier timing of inflection (**eTable 9**). However, the associations of gestational hypertension or preeclampsia with CVH trajectory parameters were attenuated to non-significance, likely due to the decrease in sample size.

Sensitivity analyses for behavioral CVH

Results for behavioral CVH were consistent with our main analyses. Specifically, children who were weaned or formula-fed only (vs. breastfeeding) in the first 6 months exhibited lower behavioral CVH from childhood to adolescence (**eTable 10**) as well as slower behavioral CVH gain before inflection and later timing of inflection (**eTable 11**).

Sensitivity analyses for biological CVH

Results for biological CVH were largely similar to the main analyses. Specifically, pre-pregnancy overweight or obesity (vs. healthy or underweight), excessive (vs. adequate) GWG, and smoking during pregnancy (vs. never) were each associated with lower biological CVH from childhood to adolescence (**eTable 12**). However, the associations of isolated hyperglycemia (vs. normal glucose tolerance) and gestational hypertension or preeclampsia (vs. normal blood pressure) with the rate of biological CVH decline after inflection were attenuated to non-significance (**eTable 13**), likely due to the decrease in sample size. Additionally, we noted that pre-pregnancy obesity (vs. healthy or underweight) was associated with faster CVH decline after inflection, which was different from the results in the main analysis. While we have no clear explanation, we speculate that the difference in findings could be potentially explained by residual confounding by unmeasured factors (e.g., maternal diet).

eTable 1: Life's Essential 8 scoring algorithm for calculating cardiovascular health (CVH) scores for each CVH metric from early childhood to late adolescence.

CVH metric	Method of measurement	Scoring algorithm
Diet	Healthy Eating Index 2015 derived from food frequency questionnaires or Automated Self-Administered 24-hour (ASA-24) Dietary Recall	**100 points**: ≥90th percentile<br>**80 points**: 75th – <90th percentile<br>**50 points**: 50th – 74th percentile<br>**25 points**: 25th – 49th percentile<br>**0 points**: 1st – 24th percentile
Nicotine exposure	Self-reported history of cigarette use, inhaled nicotine delivery system use, or mother-reported child exposure to indoor tobacco smoke	**100 points**: Never tried<br>**80 points**: Smoker in home<br>**50 points**: Tried any nicotine product, but >30 days ago<br>**25 points**: Currently using inhaled nicotine delivery system<br>**0 points**: Current combustible use (any within 30 days)
Physical activity	Mother- or self-reported minutes/week of light-to-moderate and vigorous physical activity	**<6 years**<br>**100 points:** ≥180 mins/week<br>**90 points:** 150–179 mins/week<br>**80 points:** 120–149 mins/week<br>**70 points:** 90–119 mins/week<br>**50 points:** 60–89 mins/week<br>**25 points:** 1–59 mins/week<br>**0 points:** 0 mins/week<br>**≥6 years**<br>**100 points**: ≥420 mins/week<br>**90 points**: 360–419 mins/week<br>**80 points**: 300–359 mins/week<br>**60 points**: 240–299 mins/week<br>**40 points**: 120–239 mins/week<br>**20 points**: 1–119 mins/week<br>**0 points**: 0 mins/week
Sleep duration	Mother- or self-reported hours of sleep in a usual 24-hour period	**100 points**: Age-appropriate optimal range (1-<3y: 11-14hr, 3-<6y: 10-13hr, 6-<13y: 9-12hr, 13-18y: 8-10hr)<br>**90 points**: <1hr above optimal range<br>**70 points**: <1hr below optimal range<br>**40 points**: 1–<2hr below or ≥1hr above optimal<br>**20 points**: 2–<3hr below optimal range<br>**0 points**: ≥3hr below optimal range
Body mass index (BMI)	Age- and sex-specific BMI percentiles	**100 points**: 5th – <85th percentile<br>**70 points**: 85th – <95th percentile<br>**30 points**: 95th percentile – <120% of the 95th percentile<br>**15 points**: 120% of the 95th percentile – <140% of the 95th percentile<br>**0 points**: ≥140% of the 95th percentile
Blood pressure (BP)	Age-, sex-, and height-specific BP percentiles	**100 points**: BP <90th percentile<br>**75 points**: BP ≥90th – <95th percentile, or ≥120/80 mmHg – <95th percentile, whichever is lower<br>**50 points**: BP ≥95th – <95th percentile + 12 mmHg, or 130/80 – 139/89 mmHg, whichever is lower<br>**25 points**: BP ≥95th percentile + 12 mmHg, or ≥140/90 mm Hg, whichever is lower<br>**0 points**: SBP ≥160 or ≥95th percentile + 30 mmHg SBP whichever is lower; and/or DBP ≥100 or ≥95th percentile + 20 mmHg DBP
Fasting glucose	Fasting blood glucose (FBG, mg/dL)	**100 points**: No history of diabetes and FBG <100 mg/dL, or HbA1c < 5.7%

© 2025 Aris IM et al. *JAMA Network Open*.

		**60 points**: No diabetes and FBG 100–125 mg/dL, or HbA1c 5.7–6.4%<br>**40 points**: Diabetes with HbA1c <7.0%<br>**30 points**: Diabetes with HbA1c 7.0–7.9%<br>**20 points**: Diabetes with HbA1c 8.0–8.9%<br>**10 points**: Diabetes with Hb A1c 9.0–9.9%<br>**0 points**: Diabetes with HbA1c ≥10.0%
Non-HDL cholesterol	Difference between plasma total cholesterol and HDL-cholesterol (mg/dL)	**100 points**: <100 mg/dL<br>**60 points**: 100–119 mg/dL<br>**40 points**: 120–144 mg/dL<br>**20 points**: 145–189 mg/dL<br>**0 points**: ≥190 mg/dL

© 2025 Aris IM et al. *JAMA Network Open*.

eTable 2: Participant characteristics.

	Included (n=1,333)	Excluded (n=795)	p-value
**Maternal age at enrollment in years, mean (SD)**	32.2 (5.0)	31.1 (5.4)	<0.001
**Parity, n (%)**			0.33
. Nulliparous	648 (48.6)	369 (46.4)	
. Primiparous	685 (51.4)	426 (53.6)	
**Maternal education level at enrollment, n (%)**			<0.001
. Non-college graduate	387 (29.0)	357 (46.3)	
. College graduate	946 (71.0)	414 (53.7)	
**Annual household income at enrollment, n (%)**			<0.001
. ≤$70,000/year	431 (35.1)	297 (46.1)	
. >$70,000/year	798 (64.9)	348 (53.9)	
**Maternal race and ethnicity, n (%)**			<0.001
. Non-Hispanic Asian	65 (4.9)	55 (7.1)	
. Non-Hispanic Black	181 (13.6)	167 (21.7)	
. Non-Hispanic Other [(i.e., American Indian, Alaskan Native, Pacific Islander, or Other race (unspecified)]	50 (3.8)	33 (4.3)	
. Non-Hispanic White	959 (71.9)	440 (57.1)	
. Hispanic	78 (5.9)	76 (9.9)	
**Pre-pregnancy Body Mass Index, n (%)**			0.08
. Healthy + Underweight	843 (63.2)	476 (61.1)	
. Overweight	298 (22.4)	162 (20.8)	
. Obesity	192 (14.4)	141 (18.1)	
**Gestational Weight Gain, n (%)**			0.15
. Inadequate	162 (12.2)	111 (15.2)	
. Adequate	414 (31.1)	218 (29.8)	
. Excessive	757 (56.8)	402 (55.0)	
**Hypertenstive Disorders of Pregnancy, n (%)**			0.35
. Normal blood pressure	1,171 (87.8)	674 (89.9)	
. Gestational hypertension or Pre-eclampsia	142 (10.7)	68 (9.0)	
. Chronic Hypertension	20 (1.5)	8 (1.1)	
**Gestational Glucose Tolerance, n (%)**			
. Normal glucose tolerance	1,114 (83.6)	592 (80.7)	
. Isolated hyperglycemia	111 (8.3)	69 (9.4)	
. Impaired glucose tolerance	42 (3.2)	22 (3.0)	
. Gestational Diabetes	66 (4.9)	51 (6.9)	
**Prenatal Smoking, n (%)**			<0.001
. Never	929 (69.7)	514 (66.4)	
. Before pregnancy	266 (19.9)	132 (17.1)	
. During pregnancy	138 (10.4)	128 (16.5)	
**Child sex at birth, n (%)**			0.56
. Male	680 (51.0)	416 (52.3)	
. Female	653 (49.0)	379 (47.7)	
**Gestational age at delivery in weeks, mean (SD)**	39.5 (1.7)	39.2 (2.4)	<0.001
**Birth weight-for-gestational-age z-score, mean (SD)**	0.2 (1.0)	0.1 (1.0)	0.03
**Breastfeeding Initiation, n (%)**			<0.001
. Yes	1,193 (89.5)	620 (81.9)	
. No	140 (10.5)	137 (18.1)	
**Infant feeding type in the first 6 months, n (%)**			<0.001
. Breastfeeding	345 (25.9)	82 (20.2)	
. Weaned	492 (36.9)	187 (45.9)	
. Mixed	339 (25.4)	78 (19.2)	
. Formula	157 (11.8)	60 (14.7)	
**Age in years at infancy visit, median (range)**	0.5 (0.4, 0.8)	0.5 (0.4, 0.8)	0.08
**Age in years at early childhood visit, median (range)**	3.2 (2.8, 5.9)	3.2 (2.9, 6.2)	0.17
**Age in years at mid-childhood visit, median (range)**	7.7 (6.6, 10.9)	8.0 (6.8, 10.6)	<0.001
**Age in years at early adolescent visit, median (range)**	12.9 (11.9, 16.5)	13.2 (12.0, 16.6)	0.01
**Age in years at late adolescent visit, median (range)**	17.5 (15.9, 20.1)	17.7 (15.4, 20.1)	0.22

© 2025 Aris IM et al. *JAMA Network Open*.

eTable 3: Trajectory parameters for overall, behavioral, and biological CVH in males and females (n=1,333).

	**Male N = 680**	**Female N = 653**
**Intercept of CVH trajectory**		
. Overall CVH score	77.2 (7.1)	82.2 (5.9)
. Behavioral CVH score	75.7 (3.1)	78.4 (2.7)
. Biological CVH score	78.6 (26.0)	89.1 (21.2)
**Slope of CVH trajectory before inflection point (points/year)**		
. Overall CVH score	1.2 (1.2)	0.4 (1.1)
. Behavioral CVH score	1.1 (1.0)	0.6 (1.0)
. Biological CVH score	1.5 (3.3)	0.1 (2.9)
**Timing of inflection point (years)**		
. Overall CVH score	10.2 (0.7)	10.0 (0.6)
. Behavioral CVH score	9.8 (0.3)	9.7 (0.3)
. Biological CVH score	8.4 (0.3)	8.1 (0.3)
**Slope of CVH trajectory after inflection point (points/year)**		
. Overall CVH score	-1.4 (1.1)	-1.1 (1.0)
. Behavioral CVH score	-2.0 (1.4)	-1.6 (1.4)
. Biological CVH score	-0.1 (0.8)	0.1 (0.8)

All values reflect mean and standard deviation.

eTable 4: Association of pre- and perinatal factors with projected behavioral cardiovascular health (CVH) scores at 3, 8, 13, and 18 years (n=1,310).

	β [95% CI][a]			
	**3-years**	**8-years**	**13-years**	**18-years**
**Pre-pregnancy Body Mass Index[b]**				
. Healthy + Underweight	ref	ref	ref	ref
. Overweight	-0.1 [-0.4, 0.1]	-0.2 [-1.0, 0.6]	-0.1 [-0.6, 0.4]	0.1 [-0.5, 0.7]
. Obesity	0.0 [-0.3, 0.3]	-0.3 [-1.2, 0.7]	-0.2 [-0.8, 0.4]	0.2 [-0.6, 0.9]
**Gestational Weight Gain[b]**				
. Adequate	ref	ref	ref	ref
. Inadequate	0.0 [-0.3, 0.4]	0.0 [-1.0, 1.1]	0.0 [-0.6, 0.7]	-0.3 [-1.1, 0.6]
. Excessive	-0.0 [-0.3, 0.2]	0.3 [-0.4, 1.0]	0.3 [-0.1, 0.7]	-0.1 [-0.7, 0.4]
**Hypertenstive Disorders of Pregnancy[c]**				
. Normal blood pressure	ref	ref	ref	ref
. Gestational hypertension or Pre-eclampsia	0.3 [-0.0, 0.7]	0.9 [-0.1, 1.9]	0.6 [-0.1, 1.2]	-0.2 [-1.0, 0.7]
. Chronic Hypertension	-0.1[-0.9, 0.8]	0.8 [-1.8, 3.3]	0.5 [-1.1, 2.1]	-0.8 [-2.8, 1.3]
**Gestational Glucose Tolerance[c]**				
. Normal glucose tolerance	ref	ref	ref	ref
. Isolated hyperglycemia	-0.1 [-0.4, 0.3]	-0.6 [-1.7, 0.5]	-0.4 [-1.1, 0.3]	0.3 [-0.6, 1.2]
. Impaired glucose Tolerance	0.5 [-0.1, 1.1]	1.5 [-0.3, 3.3]	0.9 [-0.2, 2.0]	0.1 [-1.4, 1.5]
. Gestational Diabetes	0.2 [-0.3, 0.7]	0.9 [-0.5, 2.4]	0.6 [-0.3, 1.5]	-0.2 [-1.4, 0.9]
**Prenatal Smoking[d]**				
. Never	ref	ref	ref	ref
. Before pregnancy	-0.1 [-0.3, 0.2]	-0.2 [-1.0, 0.6]	-0.1 [-0.6, 0.4]	0.1 [-0.5, 0.7]
. During pregnancy	**-0.6 [-1.0, -0.2]**	-0.9 [-2.0, 0.2]	-0.5 [-1.2, 0.2]	-0.1 [-1.0, 0.8]
**Breastfeeding Initiation[e]**				
. Yes	ref	ref	ref	ref
. No	-0.3 [-0.7, 0.1]	-0.7 [-1.8, 0.3]	-0.4 [-1.1, 0.2]	0.1 [-0.7, 1.0]
**Infant feeding type in the first 6 months[e]**				
. Breastfeeding	ref	ref	ref	ref
. Weaned	**-0.4 [-0.7, -0.2]**	**-0.9 [-1.8, -0.1]**	**-0.7 [-1.2, -0.2]**	-0.1 [-0.8, 0.5]
. Mixed	-0.2 [-0.5, 0.1]	-0.4 [-1.3, 0.5]	-0.4 [-0.9, 0.2]	-0.2 [-0.9, 0.5]
. Formula	**-0.6 [-1.0, -0.2]**	**-1.6 [-2.7, -0.4]**	**-1.1[-1.8, -0.4]**	0.1 [-0.8, 1.0]

[a] All β-estimates represent the mean difference in CVH score at 3-, 8-, 13-, or 18-years when comparing one category of the pre- or perinatal factor to its corresponding reference category. Bolded values reflect p<0.05.

[b] Adjusted for maternal age at enrollment, parity, race and ethnicity, education level, household income, and prenatal smoking.

[c] Adjusted for maternal age at enrollment, parity, race and ethnicity, education level, household income, prenatal smoking, and pre-pregnancy body mass index.

[d] Adjusted for maternal age at enrollment, parity, race and ethnicity, education level, and household income.

[e] Adjusted for maternal age at enrollment, parity, race and ethnicity, education level, household income, prenatal smoking, pre-pregnancy body mass index, gestational age at delivery, and birth weight-for-gestational-age z-score.

© 2025 Aris IM et al. *JAMA Network Open*.

eTable 5: Association of pre- and perinatal factors with behavioral cardiovascular health (CVH) trajectory parameters (n=1,310).

	**β [95% CI][a]**		
	**Slope of CVH before inflection (points/year)**	**Timing of inflection point (years)**	**Slope of CVH after inflection (points/year)**
**Pre-pregnancy Body Mass Index[b]**			
. Healthy + Underweight	ref	ref	ref
. Overweight	0.0 [-0.2, 0.1]	0.0 [0.0, 0.1]	0.1 [-0.1, 0.2]
. Obesity	-0.1 [-0.2, 0.1]	0.0 [-0.1, 0.1]	0.1 [-0.2, 0.3]
**Gestational Weight Gain[b]**			
. Adequate	ref	ref	ref
. Inadequate	0.0 [-0.2, 0.2]	0.0 [-0.1, 0.1]	0.0 [-0.3, 0.2]
. Excessive	0.1 [-0.0, 0.2]	0.0 [0.0, 0.0]	-0.1 [-0.3, 0.1]
**Hypertenstive Disorders of Pregnancy[c]**			
. Normal blood pressure	ref	ref	ref
. Gestational hypertension or Pre-eclampsia	0.1 [-0.1, 0.3]	0.0 [-0.1, 0.0]	-0.1 [-0.4, 0.1]
. Chronic Hypertension	0.2 [-0.3, 0.6]	0.0 [-0.1, 0.2]	-0.3 [-0.9, 0.4]
**Gestational Glucose Tolerance[c]**			
. Normal glucose tolerance	ref	ref	ref
. Isolated hyperglycemia	-0.1 [-0.3, 0.1]	0.0 [-0.1, 0.1]	0.1 [-0.1, 0.4]
. Impaired glucose tolerance	0.2 [-0.1, 0.5]	-0.1 [-0.2, 0.0]	-0.2 [-0.6, 0.3]
. Gestational Diabetes	0.1 [-0.1, 0.4]	0.0 [-0.1, 0.1]	-0.2 [-0.5, 0.2]
**Prenatal Smoking[d]**			
. Never	ref	ref	ref
. Before pregnancy	0.0 [-0.2, 0.1]	0.0 [0.0, 0.1]	0.0 [-0.2, 0.2]
. During pregnancy	-0.1 [-0.3, 0.1]	0.1 [0.0, 0.1]	0.1 [-0.2, 0.3]
**Breastfeeding Initiation[e]**			
. Yes	ref	ref	ref
. No	-0.1 [-0.3, 0.1]	0.1 [0.0, 0.1]	0.1 [-0.1, 0.4]
**Infant feeding type in the first 6 months[e]**			
. Breastfeeding	ref	ref	ref
. Weaned	-0.1 [-0.3, 0.0]	**0.1 [0.0, 0.1]**	0.1 [-0.1, 0.3]
. Mixed	-0.0 [-0.2, 0.1]	0.0 [0.0, 0.1]	0.0 [-0.2, 0.3]
. Formula	**-0.2 [-0.4, 0.0]**	**0.1 [0.0, 0.2]**	0.2 [-0.1, 0.5]

[a] All β-estimates represent the mean difference in CVH parameters (i.e., slope of CVH before inflection, timing of inflection point, or slope of CVH after inflection) when comparing one category of the pre- or perinatal factor to its corresponding reference category. Bolded values reflect $p<0.05$.

[b] Adjusted for maternal age at enrollment, parity, race and ethnicity, education level, household income, and prenatal smoking.

[c] Adjusted for maternal age at enrollment, parity, race and ethnicity, education level, household income, prenatal smoking, and pre-pregnancy body mass index.

[d] Adjusted for maternal age at enrollment, parity, race and ethnicity, education level, and household income.

[e] Adjusted for maternal age at enrollment, parity, race and ethnicity, education level, household income, prenatal smoking, pre-pregnancy body mass index, gestational age at delivery, and birth weight-for-gestational-age z-score.

© 2025 Aris IM et al. *JAMA Network Open*.

eTable 6: Association of pre- and perinatal factors with projected biological cardiovascular health scores (CVH) at 3, 8, 13, and 18 years (n=1,286).

	β [95% CI][a]			
	**3-years**	**8-years**	**13-years**	**18-years**
**Pre-pregnancy Body Mass Index[b]**				
. Healthy + Underweight	ref	ref	ref	ref
. Overweight	**-4.0 [-6.2, -1.9]**	**-1.9 [-2.9, -0.9]**	**-1.1 [-1.7, -0.6]**	-0.2 [-0.5, 0.0]
. Obesity	**-7.0 [-9.6, -4.3]**	**-4.2 [-5.4, -2.9]**	**-2.1 [-2.8, -1.4]**	**-0.4 [-0.7, -0.1]**
**Gestational Weight Gain[b]**				
. Adequate	ref	ref	ref	ref
. Inadequate	-0.2 [-3.1, 2.7]	0.3 [-1.2, 1.7]	-0.1 [-0.9, 0.7]	-0.2 [-0.5, 0.2]
. Excessive	**-2.8 [-4.8, -0.8]**	-0.9 [-1.9, 0.0]	**-0.7 [-1.3, -0.2]**	**-0.5 [-0.7, -0.2]**
**Hypertenstive Disorders of Pregnancy[c]**				
. Normal blood pressure	ref	ref	ref	ref
. Gestational hypertension or Pre-eclampsia	0.7 [-2.2, 3.5]	**1.8 [0.4, 3.2]**	**1.2 [0.4, 2.0]**	0.3 [-0.1, 0.7]
. Chronic Hypertension	-3.1 [-10.4, 4.2]	-1.5 [-4.8, 1.9]	-0.7 [-2.7, 1.2]	-0.2 [-1.1, 0.7]
**Gestational Glucose Tolerance[c]**				
. Normal glucose tolerance	ref	ref	ref	ref
. Isolated hyperglycemia	-2.3 [-5.5, 0.9]	**-1.5 [-3.0, -0.0]**	-0.7 [-1.5, 0.2]	0.1 [-0.3, 0.5]
. Impaired glucose Tolerance	0.1 [-4.9, 5.1]	0.5 [-1.9, 2.9]	0.2 [-1.2, 1.6]	0.5 [-0.1, 1.1]
. Gestational Diabetes	-1.2 [-5.3, 2.9]	0.4 [-1.6, 2.3]	0.2 [-0.9, 1.3]	-0.1 [-0.6, 0.4]
**Prenatal Smoking[d]**				
. Never	ref	ref	ref	ref
. Before pregnancy	-1.3 [-3.5, 1.0]	**-1.6 [-2.7, -0.6]**	**-0.9 [-1.5, -0.2]**	-0.2 [-0.5, 0.0]
. During pregnancy	**-6.0 [-9.0, -2.9]**	**-2.8 [-4.2, -1.3]**	**-1.3 [-2.2, -0.5]**	0.0 [-0.3, 0.4]
**Breastfeeding Initiation[e]**				
. Yes	ref	ref	ref	ref
. No	-1.0 [-4.0, 2.0]	-0.1 [-1.4, 1.3]	-0.2 [-1.0, 0.6]	-0.3 [-0.7, 0.0]
**Infant feeding type in the first 6 months[e]**				
. Breastfeeding	ref	ref	ref	ref
. Weaned	-0.9 [-3.3, 1.4]	-0.5 [-1.6, 0.6]	-0.5 [-1.2, 0.1]	**-0.3 [-0.6, -0.0]**
. Mixed	-2.2 [-4.6, 0.2]	-0.1 [-1.3, 1.1]	-0.3 [-0.9, 0.4]	-0.2 [-0.5, 0.2]
. Formula	-2.3 [-5.5, 0.9]	-0.1 [-1.6, 1.4]	-0.3 [-1.2, 0.6]	-0.4 [-0.8, 0.0]

[a] All β-estimates represent the mean difference in CVH score at 3-, 8-, 13-, or 18-years when comparing one category of the pre- or perinatal factor to its corresponding reference category. Bolded values reflect p<0.05.

[b] Adjusted for maternal age at enrollment, parity, race and ethnicity, education level, household income, and prenatal smoking.

[c] Adjusted for maternal age at enrollment, parity, race and ethnicity, education level, household income, prenatal smoking, and pre-pregnancy body mass index.

[d] Adjusted for maternal age at enrollment, parity, race and ethnicity, education level, and household income.

[e] Adjusted for maternal age at enrollment, parity, race and ethnicity, education level, household income, prenatal smoking, pre-pregnancy body mass index, gestational age at delivery, and birth weight-for-gestational-age z-score.

© 2025 Aris IM et al. *JAMA Network Open*.

eTable 7: Association of pre- and perinatal factors with biological cardiovascular health (CVH) trajectory parameters (n=1,286).

	**β [95% CI][a]**		
	**Slope of CVH before inflection (points/year)**	**Timing of inflection point (years)**	**Slope of CVH after inflection (points/year)**
**Pre-pregnancy Body Mass Index[b]**			
. Healthy + Underweight	ref	ref	ref
. Overweight	0.2 [-0.3, 0.6]	-0.0 [-0.1, 0.0]	**0.2 [0.1, 0.3]**
. Obesity	0.3 [-0.2, 0.8]	-0.1 [-0.1, 0.0]	**0.4 [0.2, 0.5]**
**Gestational Weight Gain[b]**			
. Adequate	ref	ref	ref
. Inadequate	0.3 [-0.3, 0.9]	0.0 [0.0, 0.1]	-0.1 [-0.2, 0.1]
. Excessive	0.4 [0.0, 0.8]	0.0 [0.0, 0.1]	0.1 [-0.0, 0.1]
**Hypertenstive Disorders of Pregnancy[c]**			
. Normal blood pressure	ref	ref	ref
. Gestational hypertension or Pre-eclampsia	0.4 [-0.2, 1.0]	0.1 [0.0, 0.1]	**-0.2 [-0.3, -0.1]**
. Chronic Hypertension	0.2 [-1.2, 1.7]	-0.0 [-0.2, 0.1]	0.2 [-0.2, 0.5]
**Gestational Glucose Tolerance[c]**			
. Normal glucose tolerance	ref	ref	ref
. Isolated hyperglycemia	0.0 [-0.6, 0.7]	0.0 [-0.1, 0.1]	**0.2 [0.0, 0.3]**
. Impaired glucose tolerance	0.1 [-0.9, 1.1]	0.0 [-0.1, 0.2]	-0.1 [-0.3, 0.1]
. Gestational Diabetes	0.4 [-0.4, 1.3]	0.0 [-0.1, 0.1]	-0.1 [-0.3, 0.1]
**Prenatal Smoking[d]**			
. Never	ref	ref	ref
. Before pregnancy	-0.1 [-0.6, 0.3]	0.0 [-0.1, 0.0]	**0.2 [0.1, 0.3]**
. During pregnancy	0.4 [-0.2, 1.0]	0.0 [-0.1, 0.1]	**0.2 [0.1, 0.4]**
**Breastfeeding Initiation[e]**			
. Yes	ref	ref	ref
. No	-0.1 [-0.6, 0.6]	0.0 [-0.1, 0.0]	0.0 [-0.1, 0.2]
**Infant feeding type in the first 6 months[e]**			
. Breastfeeding	ref	ref	ref
. Weaned	0.1 [-0.4, 0.5]	0.0 [-0.1, 0.0]	0.1 [-0.1, 0.2]
. Mixed	0.4 [-0.1, 0.8]	0.0 [-0.0, 0.1]	0.0 [-0.1, 0.1]
. Formula	0.3 [-0.3, 1.0]	0.0[-0.1, 0.1]	0.0 [-0.1, 0.2]

[a] All β-estimates represent the mean difference in CVH parameters (i.e., slope of CVH before inflection, timing of inflection point, or slope of CVH after inflection) when comparing one category of the pre- or perinatal factor to its corresponding reference category. Bolded values reflect p<0.05.

[b] Adjusted for maternal age at enrollment, parity, race and ethnicity, education level, household income, and prenatal smoking.

[c] Adjusted for maternal age at enrollment, parity, race and ethnicity, education level, household income, prenatal smoking, and pre-pregnancy body mass index.

[d] Adjusted for maternal age at enrollment, parity, race and ethnicity, education level, and household income.

[e] Adjusted for maternal age at enrollment, parity, race and ethnicity, education level, household income, prenatal smoking, pre-pregnancy body mass index, gestational age at delivery, and birth weight-for-gestational-age z-score.

© 2025 Aris IM et al. *JAMA Network Open*.

eTable 8: Sensitivity analyses for the association of pre- and perinatal factors with projected overall cardiovascular health (CVH) scores at 3, 8, 13, and 18 years in a subset of children with all available CVH metrics at each life stage (n=1,079).

	**β [95% CI][a]**			
	**3-years**	**8-years**	**13-years**	**18-years**
**Pre-pregnancy Body Mass Index[b]**				
. Healthy + Underweight	ref	ref	ref	ref
. Overweight	**-0.7 [-1.3, -0.1]**	-0.4 [-1.1, 0.3]	-0.4 [-0.8, 0.0]	**-0.4 [-0.8, -0.0]**
. Obesity	**-0.8 [-1.6, -0.1]**	**-1.3 [-2.2, -0.5]**	**-1.1 [-1.7, -0.6]**	**-0.6 [-1.1, -0.2]**
**Gestational Weight Gain[b]**				
. Adequate	ref	ref	ref	ref
. Inadequate	0.1 [-0.7, 0.9]	0.6 [-0.3, 1.6]	**0.7 [0.1, 1.3]**	0.0 [-0.4, 0.5]
. Excessive	-0.5 [-1.0, 0.1]	-0.1 [-0.7, 0.5]	-0.1 [-0.5, 0.3]	-0.1 [-0.4, 0.2]
**Hypertenstive Disorders of Pregnancy[c]**				
. Normal blood pressure	ref	ref	ref	ref
. Gestational hypertension or Pre-eclampsia	0.5 [-0.3, 1.3]	0.7 [-0.2, 1.6]	**0.6 [0.0, 1.2]**	0.4 [-0.1, 0.9]
. Chronic Hypertension	0.5 [-1.7, 2.7]	0.7 [-1.7, 3.2]	0.8 [-0.8, 2.4]	0.5 [-0.8, 1.8]
**Gestational Glucose Tolerance[c]**				
. Normal glucose tolerance	ref	ref	ref	ref
. Isolated hyperglycemia	0.3 [-0.6, 1.1]	-0.1 [-1.0, 0.9]	-0.2 [-0.8, 0.5]	-0.1 [-0.6, 0.4]
. Impaired glucose Tolerance	0.3 [-1.0, 1.7]	1.3 [-0.2, 2.8]	0.3 [-0.7, 1.3]	-0.1 [-1.0, 0.7]
. Gestational Diabetes	-0.2 [-1.3, 1.0]	0.7 [-0.6, 2.0]	0.7 [-0.1, 1.6]	0.3 [-0.5, 1.0]
**Prenatal Smoking[d]**				
. Never	ref	ref	ref	ref
. Before pregnancy	-0.1 [-0.7, 0.5]	-0.6 [-1.3, 0.1]	-0.4 [-0.9, 0.1]	0.1 [-0.3 ,0.4]
. During pregnancy	**-1.4 [-2.3, -0.6]**	**-1.8 [-2.8, -0.8]**	**-1.5 [-2.1, -0.8]**	-0.5 [-1.0, 0.0]
**Breastfeeding Initiation[e]**				
. Yes	ref	ref	ref	ref
. No	-0.7 [-1.6, 0.2]	-0.0 [-1.0, 1.0]	0.1 [-0.6, 0.7]	-0.2 [-0.7, 0.3]
**Infant feeding type in the first 6 months[e]**				
. Breastfeeding	ref	ref	ref	ref
. Weaned	**-0.8 [-1.4, -0.1]**	**-0.8 [-1.5, -0.0]**	**-0.7 [-1.1, -0.2]**	**-0.5 [-0.9, -0.2]**
. Mixed	**-0.7 [-1.4, -0.0]**	-0.3 [-1.1, 0.4]	-0.4 [-0.9, 0.1]	**-0.6 [-1.0, -0.2]**
. Formula	**-1.2 [-2.1, -0.2]**	-0.5 [-1.6, 0.5]	-0.4 [-1.1, 0.3]	**-0.7 [-1.2, -0.1]**

[a] All β-estimates represent the mean difference in CVH score at 3-, 8-, 13-, or 18-years when comparing one category of the pre- or perinatal factor to its corresponding reference category. Bolded values reflect p<0.05.

[b] Adjusted for maternal age at enrollment, parity, race and ethnicity, education level, household income, and prenatal smoking.

[c] Adjusted for maternal age at enrollment, parity, race and ethnicity, education level, household income, prenatal smoking, and pre-pregnancy body mass index.

[d] Adjusted for maternal age at enrollment, parity, race and ethnicity, education level, and household income.

[e] Adjusted for maternal age at enrollment, parity, race and ethnicity, education level, household income, prenatal smoking, pre-pregnancy body mass index, gestational age at delivery, and birth weight-for-gestational-age z-score.

© 2025 Aris IM et al. *JAMA Network Open*.

eTable 9: Sensitivity analyses for the association of pre- and perinatal factors with overall cardiovascular health (CVH) trajectory parameters in a subset of children with all available CVH metrics at each life stage (n=1,079).

	β [95% CI][a]		
	**Slope of CVH before inflection (points/year)**	**Timing of inflection point (years)**	**Slope of CVH after inflection (points/year)**
**Pre-pregnancy Body Mass Index[b]**			
. Healthy + Underweight	ref	ref	ref
. Overweight	0.0 [-0.1, 0.2]	0.0 [-0.1, 0.2]	0.0 [-0.1, 0.1]
. Obesity	-0.1 [-0.3, 0.1]	**0.2 [0.1, 0.4]**	0.1 [-0.0, 0.2]
**Gestational Weight Gain[b]**			
. Adequate	ref	ref	ref
. Inadequate	0.1 [-0.1, 0.3]	0.0 [-0.2, 0.2]	-0.1 [-0.2, 0.0]
. Excessive	0.1 [-0.1, 0.2]	-0.1 [-0.2, 0.1]	-0.0 [-0.1, 0.1]
**Hypertenstive Disorders of Pregnancy[c]**			
. Normal blood pressure	ref	ref	ref
. Gestational hypertension or Pre-eclampsia	0.0 [-0.2, 0.2]	-0.1 [-0.3, 0.1]	-0.1 [-0.2, 0.1]
. Chronic Hypertension	0.0 [-0.5, 0.6]	-0.1 [-0.6, 0.4]	-0.1 [-0.4, 0.3]
**Gestational Glucose Tolerance[c]**			
. Normal glucose tolerance	ref	ref	ref
. Isolated hyperglycemia	-0.1 [-0.3, 0.1]	0.1 [-0.1, 0.3]	0.0 [-0.1, 0.1]
. Impaired glucose tolerance	0.2 [-0.1, 0.5]	**-0.4 [-0.7, 0.0]**	-0.1 [-0.3, 0.1]
. Gestational Diabetes	0.2 [-0.1, 0.4]	-0.1 [-0.4, 0.2]	-0.1 [-0.3, 0.1]
**Prenatal Smoking[d]**			
. Never	ref	ref	ref
. Before pregnancy	-0.1 [-0.3, 0.0]	0.1 [-0.1, 0.2]	0.1 [0.0, 0.2]
. During pregnancy	-0.1 [-0.3, 0.1]	0.1 [-0.1, 0.3]	**0.2 [0.1, 0.3]**
**Breastfeeding Initiation[e]**			
. Yes	ref	ref	ref
. No	0.1 [-0.1, 0.3]	0.0 [-0.2, 0.2]	-0.0 [-0.2, 0.1]
**Infant feeding type in the first 6 months[e]**			
. Breastfeeding	ref	ref	ref
. Weaned	0.0 [-0.2, 0.2]	**0.2 [0.0, 0.3]**	0.0 [-0.1, 0.1]
. Mixed	0.1 [-0.1, 0.2]	0.1 [-0.1, 0.2]	0.0 [-0.1, 0.1]
. Formula	0.1 [-0.1, 0.3]	0.2 [-0.1, 0.4]	-0.1 [-0.2, 0.1]

[a] All β-estimates represent the mean difference in CVH parameters (i.e., slope of CVH before inflection, timing of inflection point, or slope of CVH after inflection) when comparing one category of the pre- or perinatal factor to its corresponding reference category. Bolded values reflect $p<0.05$.

[b] Adjusted for maternal age at enrollment, parity, race and ethnicity, education level, household income, and prenatal smoking.

[c] Adjusted for maternal age at enrollment, parity, race and ethnicity, education level, household income, prenatal smoking, and pre-pregnancy body mass index.

[d] Adjusted for maternal age at enrollment, parity, race and ethnicity, education level, and household income.

[e] Adjusted for maternal age at enrollment, parity, race and ethnicity, education level, household income, prenatal smoking, pre-pregnancy body mass index, gestational age at delivery, and birth weight-for-gestational-age z-score.

© 2025 Aris IM et al. *JAMA Network Open*.

eTable 10: Sensitivity analyses for the association of pre- and perinatal factors with projected behavioral cardiovascular health (CVH) scores at 3, 8, 13, and 18 years in a subset of children with all available CVH metrics at each life stage (n=1,079).

	β [95% CI][a]			
	**3-years**	**8-years**	**13-years**	**18-years**
**Pre-pregnancy Body Mass Index[b]**				
. Healthy + Underweight	ref	ref	ref	ref
. Overweight	-0.1 [-0.4, 0.1]	-0.5 [-1.3, 0.3]	-0.2 [-0.7, 0.3]	0.2 [-0.2, 0.5]
. Obesity	0.1 [-0.2, 0.3]	-0.1 [-1.1, 0.9]	-0.0 [-0.6, 0.6]	0.1 [-0.4, 0.5]
**Gestational Weight Gain[b]**				
. Adequate	ref	ref	ref	ref
. Inadequate	-0.0 [-0.3, 0.3]	0.1 [-1.0, 1.2]	0.1 [-0.6, 0.7]	-0.2 [-0.7, 0.3]
. Excessive	-0.0 [-0.2, 0.2]	0.2 [-0.6, 0.9]	0.1 [-0.3, 0.6]	0.1 [-0.2, 0.4]
**Hypertenstive Disorders of Pregnancy[c]**				
. Normal blood pressure	ref	ref	ref	ref
. Gestational hypertension or Pre-eclampsia	0.3 [-0.0, 0.6]	0.9 [-0.2, 2.0]	0.6 [-0.1, 1.2]	0.1 [-0.4, 0.6]
. Chronic Hypertension	0.3 [-0.5, 1.1]	1.2 [-1.7, 4.2]	0.8 [-0.9, 2.5]	-0.0 [-1.3, 1.3]
**Gestational Glucose Tolerance[c]**				
. Normal glucose tolerance	ref	ref	ref	ref
. Isolated hyperglycemia	0.1 [-0.2, 0.4]	-0.1 [-1.3, 1.0]	-0.0 [-0.7, 0.7]	0.2 [-0.3, 0.7]
. Impaired glucose Tolerance	-0.1 [-0.6, 0.4]	0.3 [-1.5, 2.2]	0.1 [-1.0, 1.2]	0.0 [-0.8, 0.9]
. Gestational Diabetes	0.3 [-0.2, 0.7]	0.5 [-1.1, 2.1]	0.4 [-0.6, 1.3]	0.5 [-0.2, 1.2]
**Prenatal Smoking[d]**				
. Never	ref	ref	ref	ref
. Before pregnancy	-0.1 [-0.3, 0.2]	-0.2 [-1.0, 0.6]	-0.1 [-0.6, 0.4]	0.0 -0.3, 0.4]
. During pregnancy	**-0.6 [-1.0, -0.3]**	**-1.7 [-2.9, -0.6]**	**-1.1 [-1.8, -0.4]**	-0.3 [-0.8, 0.2]
**Breastfeeding Initiation[e]**				
. Yes	ref	ref	ref	ref
. No	-0.1 [-0.4, 0.2]	-0.2 [-1.4, 1.0]	-0.0 [-0.7, 0.6]	0.0 [-0.5, 0.6]
**Infant feeding type in the first 6 months[e]**				
. Breastfeeding	ref	ref	ref	ref
. Weaned	**-0.3 [-0.6, -0.1]**	**-0.9 [-1.8, -0.1]**	**-0.7 [-1.2, -0.2]**	**-0.4 [-0.8, -0.0]**
. Mixed	-0.2 [-0.4, 0.0]	-0.7 [-1.6, 0.2]	-0.4 [-0.9, 0.1]	-0.1 [-0.5, 0.3]
. Formula	**-0.4 [-0.7, -0.0]**	-1.2 [-2.4, 0.1]	**-0.7 [-1.4, 0.0]**	-0.2 [-0.8, 0.4]

[a] All β-estimates represent the mean difference in CVH score at 3-, 8-, 13-, or 18-years when comparing one category of the pre- or perinatal factor to its corresponding reference category. Bolded values reflect p<0.05.
[b] Adjusted for maternal age at enrollment, parity, race and ethnicity, education level, household income, and prenatal smoking.
[c] Adjusted for maternal age at enrollment, parity, race and ethnicity, education level, household income, prenatal smoking, and pre-pregnancy body mass index.
[d] Adjusted for maternal age at enrollment, parity, race and ethnicity, education level, and household income.
[e] Adjusted for maternal age at enrollment, parity, race and ethnicity, education level, household income, prenatal smoking, pre-pregnancy body mass index, gestational age at delivery, and birth weight-for-gestational-age z-score.

© 2025 Aris IM et al. *JAMA Network Open*.

eTable 11: Sensitivity analyses for the association of pre- and perinatal factors with behavioral cardiovascular health (CVH) trajectory parameters in a subset of children with all available CVH metrics at each life stage (n=1,079).

	**β [95% CI][a]**		
	**Slope of CVH before inflection (points/year)**	**Timing of inflection point (years)**	**Slope of CVH after inflection (points/year)**
**Pre-pregnancy Body Mass Index[b]**			
. Healthy + Underweight	ref	ref	ref
. Overweight	-0.1 [-0.2, 0.1]	0.0 [0.0, 0.1]	0.1 [0.0, 0.2]
. Obesity	-0.0 [-0.2, 0.1]	0.0 [-0.1, 0.1]	0.0 [-0.1, 0.2]
**Gestational Weight Gain[b]**			
. Adequate	ref	ref	ref
. Inadequate	0.0 [-0.2, 0.2]	0.0 [0.0, 0.1]	0.0 [-0.2, 0.1]
. Excessive	0.0 [-0.1, 0.2]	-0.0 [-0.1, 0.0]	0.0 [-0.1, 0.1]
**Hypertenstive Disorders of Pregnancy[c]**			
. Normal blood pressure	ref	ref	ref
. Gestational hypertension or Pre-eclampsia	0.1 [-0.0, 0.3]	-0.1 [-0.1, 0.0]	-0.1 [-0.3, 0.1]
. Chronic Hypertension	0.2 [-0.3, 0.7]	-0.0 [-0.2, 0.2]	-0.2 [-0.6, 0.3]
**Gestational Glucose Tolerance[c]**			
. Normal glucose tolerance	ref	ref	ref
. Isolated hyperglycemia	-0.0 [-0.2, 0.1]	0.0 [-0.1, 0.1]	0.0 [-0.1, 0.2]
. Impaired glucose tolerance	0.1 [-0.2, 0.4]	0.0 [-0.2, 0.1]	-0.0 [-0.3, 0.3]
. Gestational Diabetes	0.0 [-0.2, 0.3]	-0.1 [-0.2, 0.0]	0.0 [-0.2, 0.3]
**Prenatal Smoking[d]**			
. Never	ref	ref	ref
. Before pregnancy	0.0 [-0.2,0.1]	0.0 [0.0, 0.1]	0.0 [-0.1, 0.1]
. During pregnancy	**-0.2 [-0.4, 0.0]**	**0.1 [0.0, 0.2]**	0.2 [0.0, 0.3]
**Breastfeeding Initiation[e]**			
. Yes	ref	ref	ref
. No	0.0 [-0.2, 0.2]	0.0 [-0.1, 0.1]	0.0 [-0.2, 0.2]
**Infant feeding type in the first 6 months[e]**			
. Breastfeeding	ref	ref	ref
. Weaned	**-0.1 [-0.3, 0.0]**	**0.1 [0.0, 0.2]**	0.1 [-0.1, 0.2]
. Mixed	-0.1 [-0.2, 0.1]	0.0 [0.0, 0.1]	0.1 [-0.1, 0.2]
. Formula	**-0.2 [-0.4, 0.0]**	**0.1 [0.0, 0.2]**	0.1 [-0.1, 0.3]

[a] All β-estimates represent the mean difference in CVH parameters (i.e., slope of CVH before inflection, timing of inflection point, or slope of CVH after inflection) when comparing one category of the pre- or perinatal factor to its corresponding reference category. Bolded values reflect $p<0.05$.

[b] Adjusted for maternal age at enrollment, parity, race and ethnicity, education level, household income, and prenatal smoking.

[c] Adjusted for maternal age at enrollment, parity, race and ethnicity, education level, household income, prenatal smoking, and pre-pregnancy body mass index.

[d] Adjusted for maternal age at enrollment, parity, race and ethnicity, education level, and household income.

[e] Adjusted for maternal age at enrollment, parity, race and ethnicity, education level, household income, prenatal smoking, pre-pregnancy body mass index, gestational age at delivery, and birth weight-for-gestational-age z-score.

© 2025 Aris IM et al. *JAMA Network Open*.

eTable 12: Sensitivity analyses for the association of pre- and perinatal factors with projected biological cardiovascular health (CVH) scores at 3, 8, 13, and 18 years in a subset of children with all available CVH metrics at each life stage (n=1,079).

	**β [95% CI][a]**			
	**3-years**	**8-years**	**13-years**	**18-years**
**Pre-pregnancy Body Mass Index[b]**				
. Healthy + Underweight	ref	ref	ref	ref
. Overweight	**-3.6 [-5.9, -1.3]**	-0.5 [-1.2, 0.3]	-0.6 [-1.2, 0.0]	**-1.8 [-3.0, -0.6]**
. Obesity	**-5.9 [-8.7, -3.0]**	**-1.3 [-2.2, -0.4]**	**-1.3 [-2.1, -0.5]**	**-3.3 [-4.7, -1.8]**
**Gestational Weight Gain[b]**				
. Adequate	ref	ref	ref	ref
. Inadequate	0.3 [-2.8, 3.5]	0.3 [-0.7, 1.3]	-0.0 [-0.9, 0.9]	-0.2 [-1.8, 1.4]
. Excessive	**-2.6 [-4.8, -0.5]**	-0.6 [-1.2, 0.1]	**-0.6 [-1.2, -0.1]**	**-1.5 [-2.6, -0.4]**
**Hypertenstive Disorders of Pregnancy[c]**				
. Normal blood pressure	ref	ref	ref	ref
. Gestational hypertension or Pre-eclampsia	2.0 [-1.1, 5.1]	0.5 [-0.5, 1.5]	0.6 [-0.3, 1.4]	0.6 [-1.0, 2.3]
. Chronic Hypertension	1.2 [-7.1, 9.5]	0.8 [-1.8, 3.4]	0.5 [-1.8, 2.8]	-0.0 [-4.3, 4.3]
**Gestational Glucose Tolerance[c]**				
. Normal glucose tolerance	ref	ref	ref	ref
. Isolated hyperglycemia	-0.7 [-4.0, 2.5]	-0.3 [-1.4, 0.7]	-0.0 [-0.9, 0.9]	-0.4 [-2.1, 1.3]
. Impaired glucose Tolerance	1.9 [-3.2, 7.0]	0.8 [-0.9, 2.4]	0.8 [-0.6, 2.2]	-0.2 [-3.0, 2.5]
. Gestational Diabetes	-2.1 [-6.6, 2.4]	0.2 [-1.2, 1.6]	0.3 [-0.9, 1.6]	-1.1 [-3.4, 1.2]
**Prenatal Smoking[d]**				
. Never	ref	ref	ref	ref
. Before pregnancy	-1.3 [-3.7, 1.2]	-0.7 [-1.4, 0.1]	-0.5 [-1.2, 0.2]	-0.3 [-1.6, 0.9]
. During pregnancy	**-3.5 [-6.8, -0.2]**	**-1.7 [-2.7, -0.7]**	**-1.3 [-2.2, -0.3]**	**-1.9 [-3.7, -0.2]**
**Breastfeeding Initiation[e]**				
. Yes	ref	ref	ref	ref
. No	-2.5 [-5.8, 0.9]	-0.3 [-1.4, 0.7]	-0.3 [-1.2, 0.6]	-1.2 [-2.9, 0.6]
**Infant feeding type in the first 6 months[e]**				
. Breastfeeding	ref	ref	ref	ref
. Weaned	-1.7 [-4.2, 0.7]	-0.1 [-0.9, 0.6]	-0.4 [-1.1, 0.3]	-0.8 [-2.1, 0.4]
. Mixed	**-2.6 [-5.1, -0.0]**	-0.1 [-0.8, 0.7]	-0.4 [-1.1, 0.3]	**-1.4 [-2.7, -0.1]**
. Formula	**-3.6 [-7.2, -0.1]**	-0.3 [-1.4, 0.8]	-0.5 [-1.5, 0.5]	**-1.8 [-3.6, 0.0]**

[a] All β-estimates represent the mean difference in CVH score at 3-, 8-, 13-, or 18-years when comparing one category of the pre- or perinatal factor to its corresponding reference category. Bolded values reflect p<0.05.
[b] Adjusted for maternal age at enrollment, parity, race and ethnicity, education level, household income, and prenatal smoking.
[c] Adjusted for maternal age at enrollment, parity, race and ethnicity, education level, household income, prenatal smoking, and pre-pregnancy body mass index.
[d] Adjusted for maternal age at enrollment, parity, race and ethnicity, education level, and household income.
[e] Adjusted for maternal age at enrollment, parity, race and ethnicity, education level, household income, prenatal smoking, pre-pregnancy body mass index, gestational age at delivery, and birth weight-for-gestational-age z-score.

© 2025 Aris IM et al. *JAMA Network Open*.

eTable 13: Sensitivity analyses for the association of pre- and perinatal factors with biological cardiovascular health (CVH) trajectory parameters in a subset of children with all available CVH metrics at each life stage (n=1,079).

	**β [95% CI][a]**		
	**Slope of CVH before inflection (points/year)**	**Timing of inflection point (years)**	**Slope of CVH after inflection (points/year)**
**Pre-pregnancy Body Mass Index[b]**			
. Healthy + Underweight	ref	ref	ref
. Overweight	**0.6 [0.2, 0.9]**	-0.1 [-0.1, 0.0]	-0.2 [-0.4, 0.0]
. Obesity	**1.0 [0.5, 1.5]**	**-0.2 [-0.2, -0.1]**	**-0.4 [-0.6, -0.2]**
**Gestational Weight Gain[b]**			
. Adequate	ref	ref	ref
. Inadequate	0.0 [-0.5, 0.5]	0.0 [-0.1, 0.1]	0.0 [-0.3, 0.2]
. Excessive	**0.4 [0.0, 0.7]**	**-0.1 [-0.1, 0.0]**	-0.1 [-0.3, 0.0]
**Hypertenstive Disorders of Pregnancy[c]**			
. Normal blood pressure	ref	ref	ref
. Gestational hypertension or Pre-eclampsia	0.1 [-0.6, 0.4]	0.0 [-0.1, 0.1]	0.0 [-0.3, 0.3]
. Chronic Hypertension	0.1 [-1.3, 1.4]	0.0 [-0.2, 0.2]	-0.1 [-0.8, 0.6]
**Gestational Glucose Tolerance[c]**			
. Normal glucose tolerance	ref	ref	ref
. Isolated hyperglycemia	0.2 [-0.4, 0.7]	0.0 [-0.1, 0.0]	0.0 [-0.3, 0.2]
. Impaired glucose tolerance	0.0 [-0.8, 0.9]	0.0 [-0.1, 0.1]	-0.1 [-0.5, 0.3]
. Gestational Diabetes	0.5 [-0.2, 1.3]	0.0 [-0.1, 0.1]	-0.3 [-0.7, 0.1]
**Prenatal Smoking[d]**			
. Never	ref	ref	ref
. Before pregnancy	0.1 [-0.3, 0.5]	-0.0 [-0.1, 0.0]	0.1 [-0.1, 0.2]
. During pregnancy	0.5 [0.0, 1.0]	-0.1 [-0.2, 0.0]	-0.1 [-0.3, 0.2]
**Breastfeeding Initiation[e]**			
. Yes	ref	ref	ref
. No	0.4 [-0.1, 1.0]	-0.1 [-0.1, 0.0]	-0.2 [-0.5, 0.1]
**Infant feeding type in the first 6 months[e]**			
. Breastfeeding	ref	ref	ref
. Weaned	0.2 [-0.2, 0.6]	0.0 [-0.1, 0.0]	-0.1 [-0.3, 0.1]
. Mixed	0.4 [0.0, 0.9]	-0.1 [-0.1, 0.0]	-0.2 [-0.4, 0.0]
. Formula	0.6 [0.0, 1.1]	-0.1 [-0.2, 0.0]	-0.3 [-0.6, 0.0]

[a] All β-estimates represent the mean difference in CVH parameters (i.e., slope of CVH before inflection, timing of inflection point, or slope of CVH after inflection) when comparing one category of the pre- or perinatal factor to its corresponding reference category. Bolded values reflect $p<0.05$.

[b] Adjusted for maternal age at enrollment, parity, race and ethnicity, education level, household income, and prenatal smoking.

[c] Adjusted for maternal age at enrollment, parity, race and ethnicity, education level, household income, prenatal smoking, and pre-pregnancy body mass index.

[d] Adjusted for maternal age at enrollment, parity, race and ethnicity, education level, and household income.

[e] Adjusted for maternal age at enrollment, parity, race and ethnicity, education level, household income, prenatal smoking, pre-pregnancy body mass index, gestational age at delivery, and birth weight-for-gestational-age z-score.

© 2025 Aris IM et al. *JAMA Network Open*.

eFigure 1: Flowchart for analytic sample.

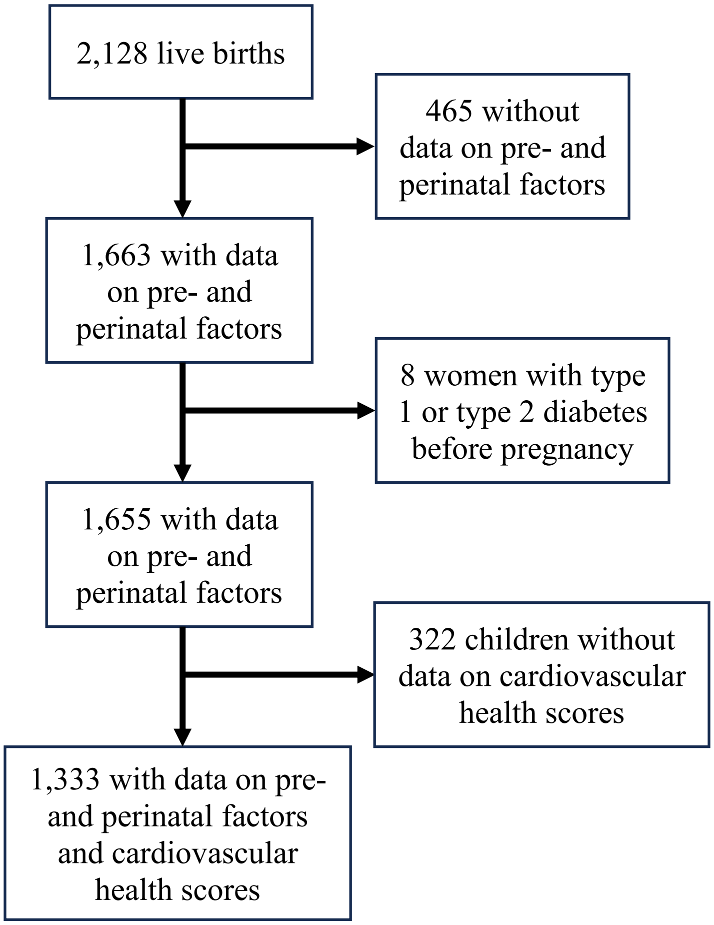

© 2025 Aris IM et al. *JAMA Network Open*.

eFigure 2: Directed acyclic graph describing the relationship of pre- and perinatal factors, covariates, and cardiovascular health in children. BMI: body mass index; BF: breastfeeding; BW-for-GA: birth weight-for-gestational-age; CVH: cardiovascular health; GWG: gestational weight gain.

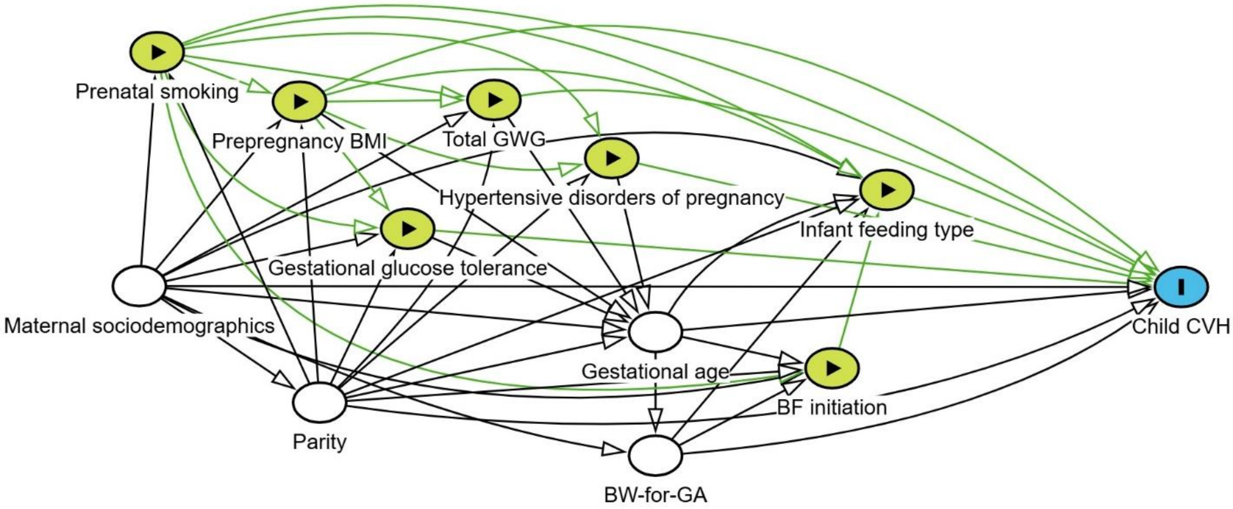

© 2025 Aris IM et al. *JAMA Network Open*.

eFigure 3: Unadjusted trajectories of behavioral cardiovascular health (CVH) scores from early childhood to late adolescence according to prepregnancy body mass index (**A**), gestational weight gain (**B**), hypertensive disorders of pregnancy (**C**), and gestational glucose tolerance (**D**). GH: gestational hypertension; PE: preeclampsia.

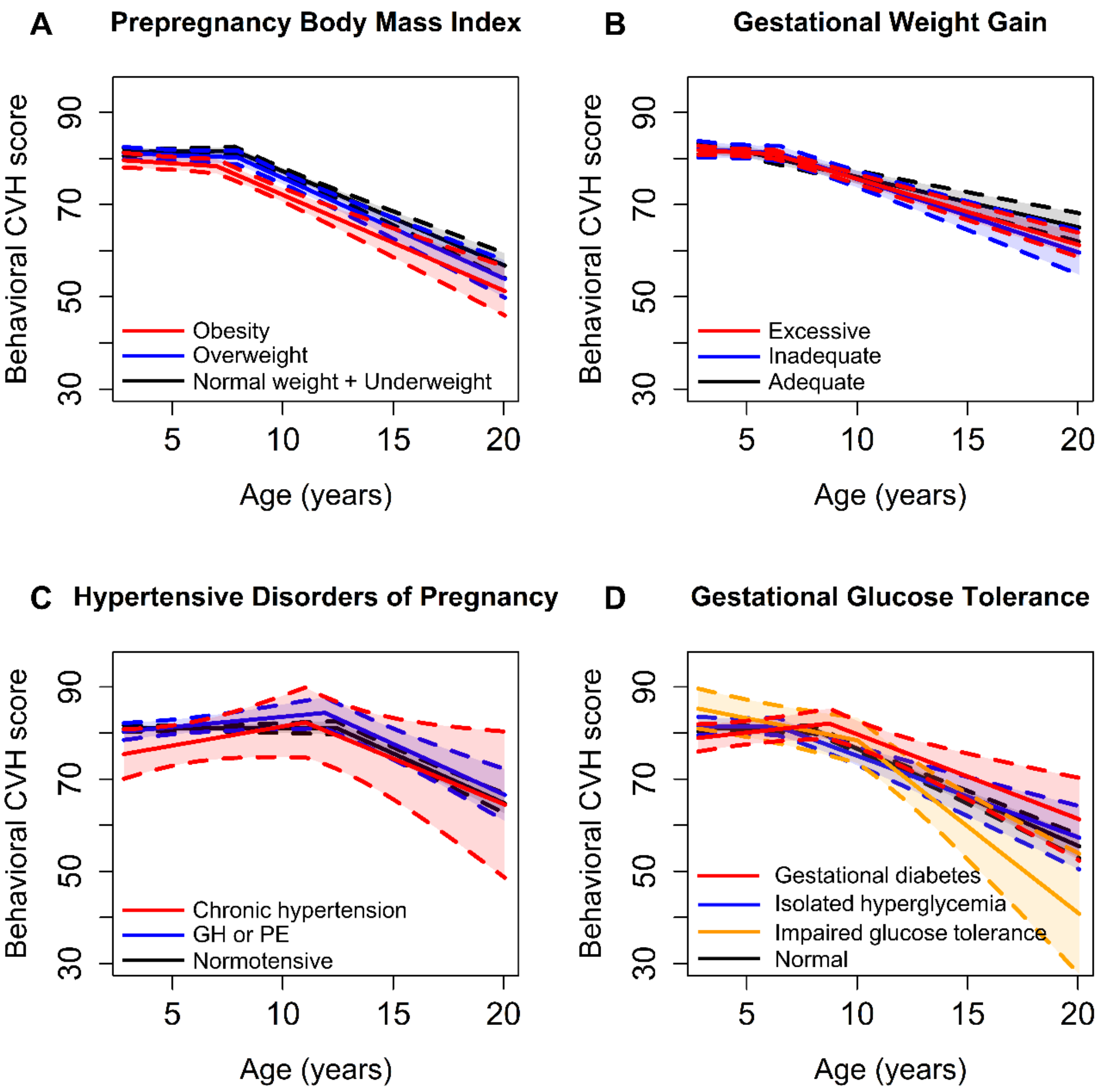

© 2025 Aris IM et al. *JAMA Network Open*.

eFigure 4: Unadjusted trajectories of behavioral cardiovascular health (CVH) score from early childhood to late adolescence according to prenatal smoking status (**A**), breastfeeding initiation (**B**), and infant feeding type in the first 6 months (**C**).

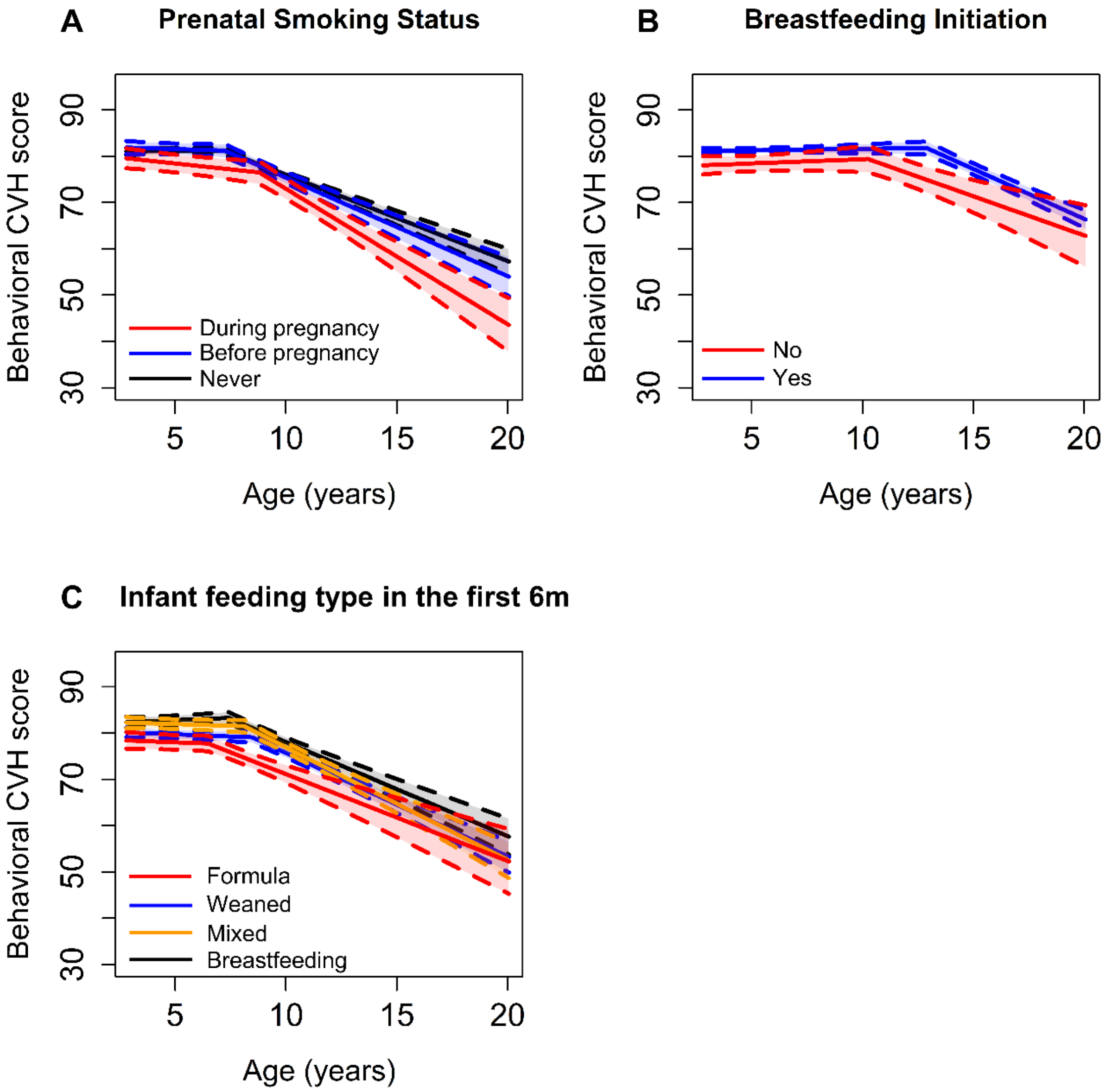

© 2025 Aris IM et al. *JAMA Network Open*.

eFigure 5: Unadjusted trajectories of biological cardiovascular health (CVH) scores from early childhood to late adolescence according to pre-pregnancy body mass index (**A**), gestational weight gain (**B**), hypertensive disorders of pregnancy (**C**), and gestational glucose tolerance (**D**) GH: gestational hypertension; PE: preeclampsia.

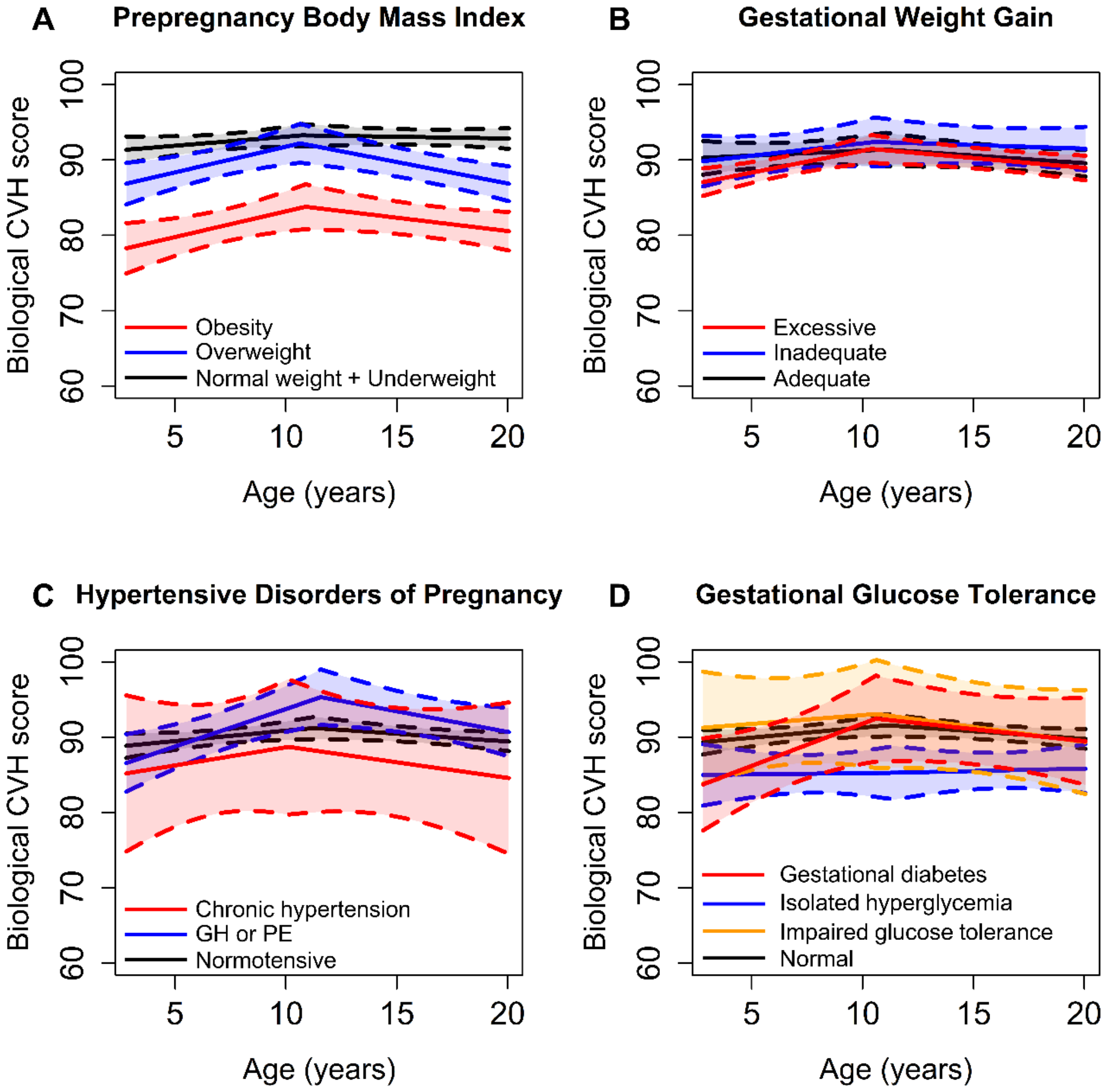

© 2025 Aris IM et al. *JAMA Network Open*.

eFigure 6: Unadjusted trajectories of biological cardiovascular health (CVH) score from early childhood to late adolescence according to prenatal smoking status (**A**), breastfeeding initiation (**B**), and infant feeding type in the first 6 months (**C**).

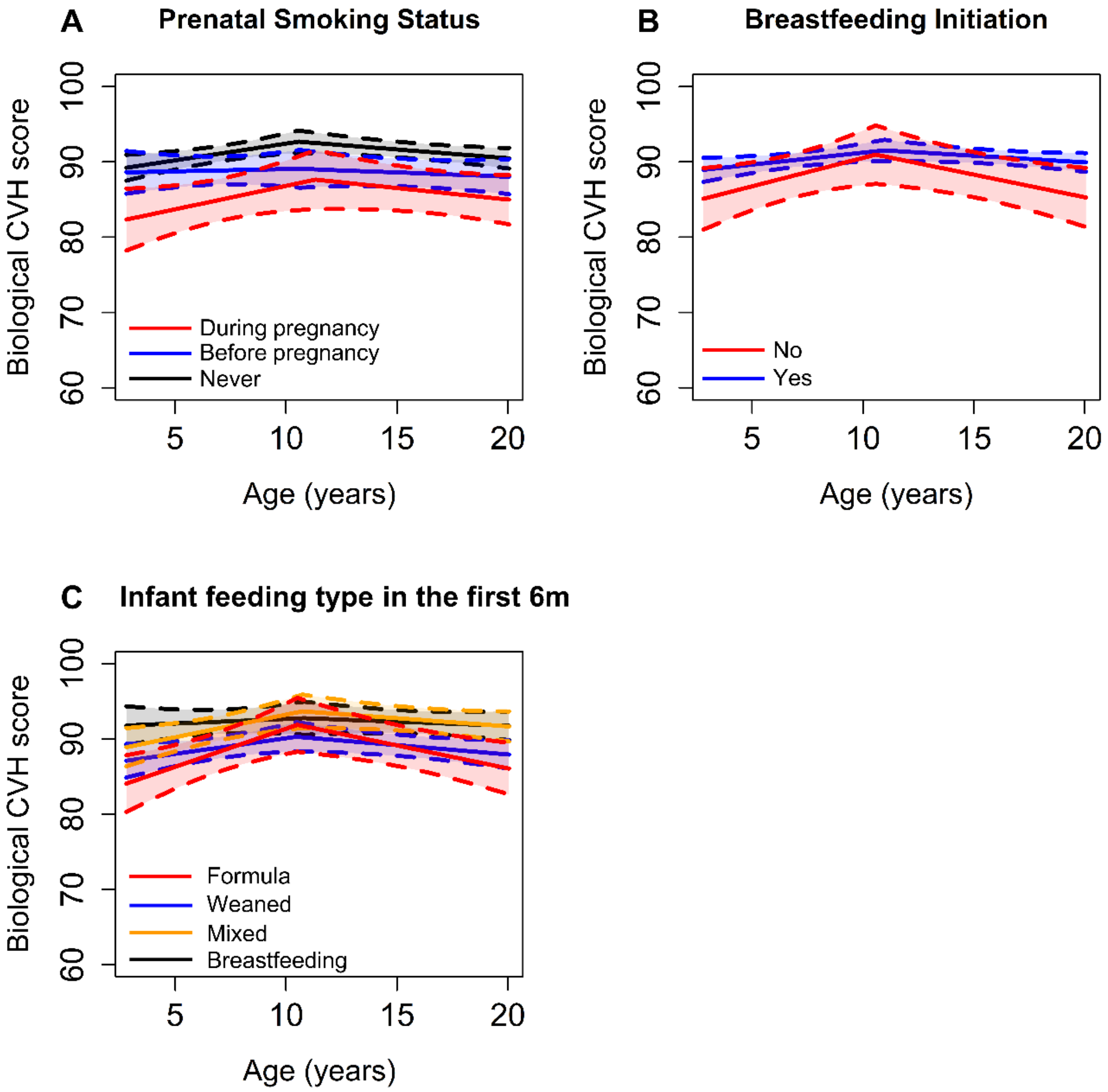

© 2025 Aris IM et al. *JAMA Network Open*.

eFigure 7: Unadjusted trajectories of overall cardiovascular health (CVH) scores from early childhood to late adolescence according to pre-pregnancy body mass index (**A**), gestational weight gain (**B**), hypertensive disorders of pregnancy (**C**), and gestational glucose tolerance (**D**). Trajectories are restricted to a subset of children (n=1,079) with all available CVH metrics at each life stage. GH: gestational hypertension; PE: preeclampsia.

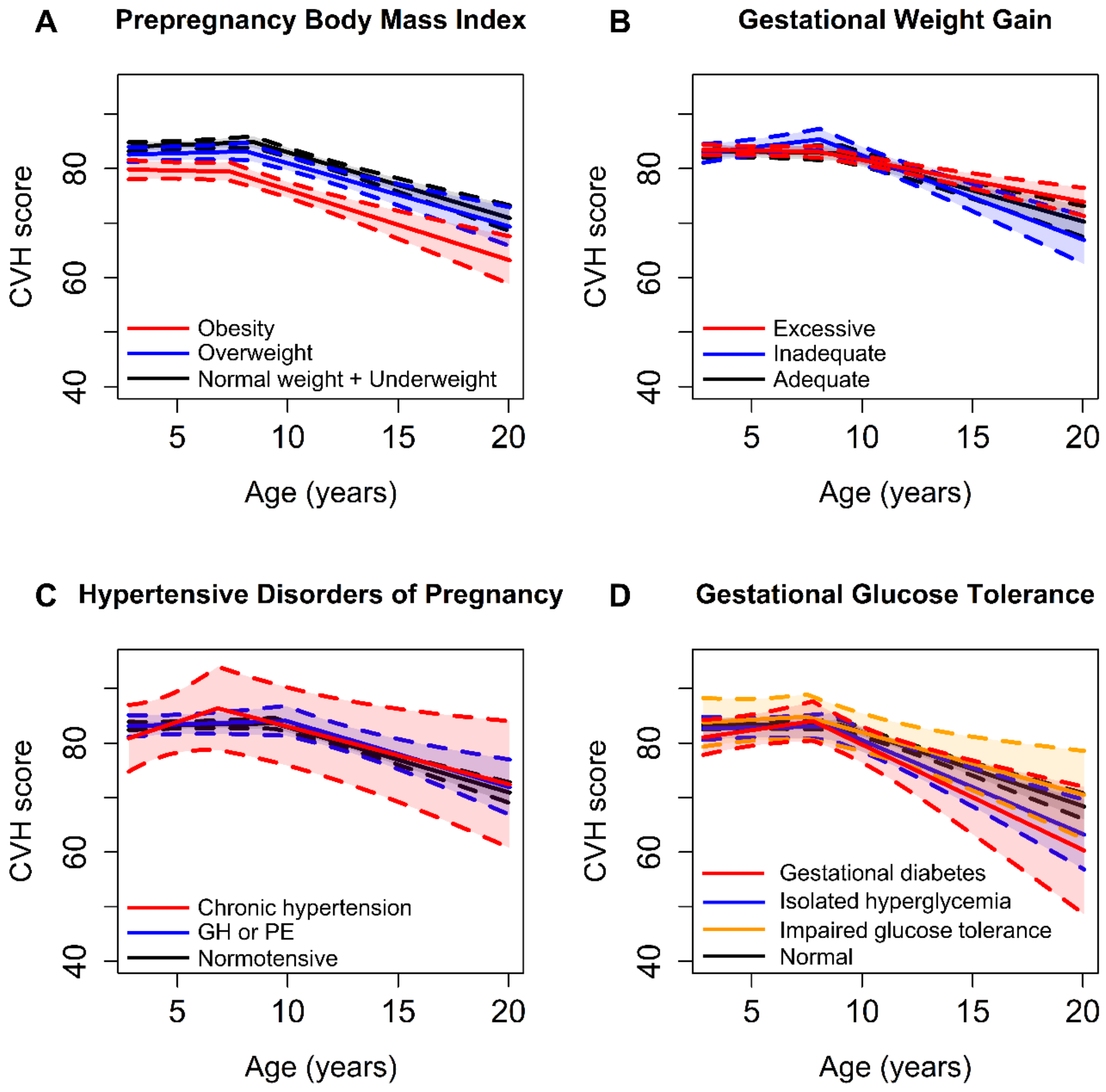

© 2025 Aris IM et al. *JAMA Network Open*.

eFigure 8: Unadjusted trajectories of overall cardiovascular health (CVH) scores from early childhood to late adolescence according to prenatal smoking status (**A**), breastfeeding initiation (**B**), and infant feeding type in the first 6 months (**C**). Trajectories are restricted to a subset of children (n=1,079) with all available CVH metrics at each life stage.

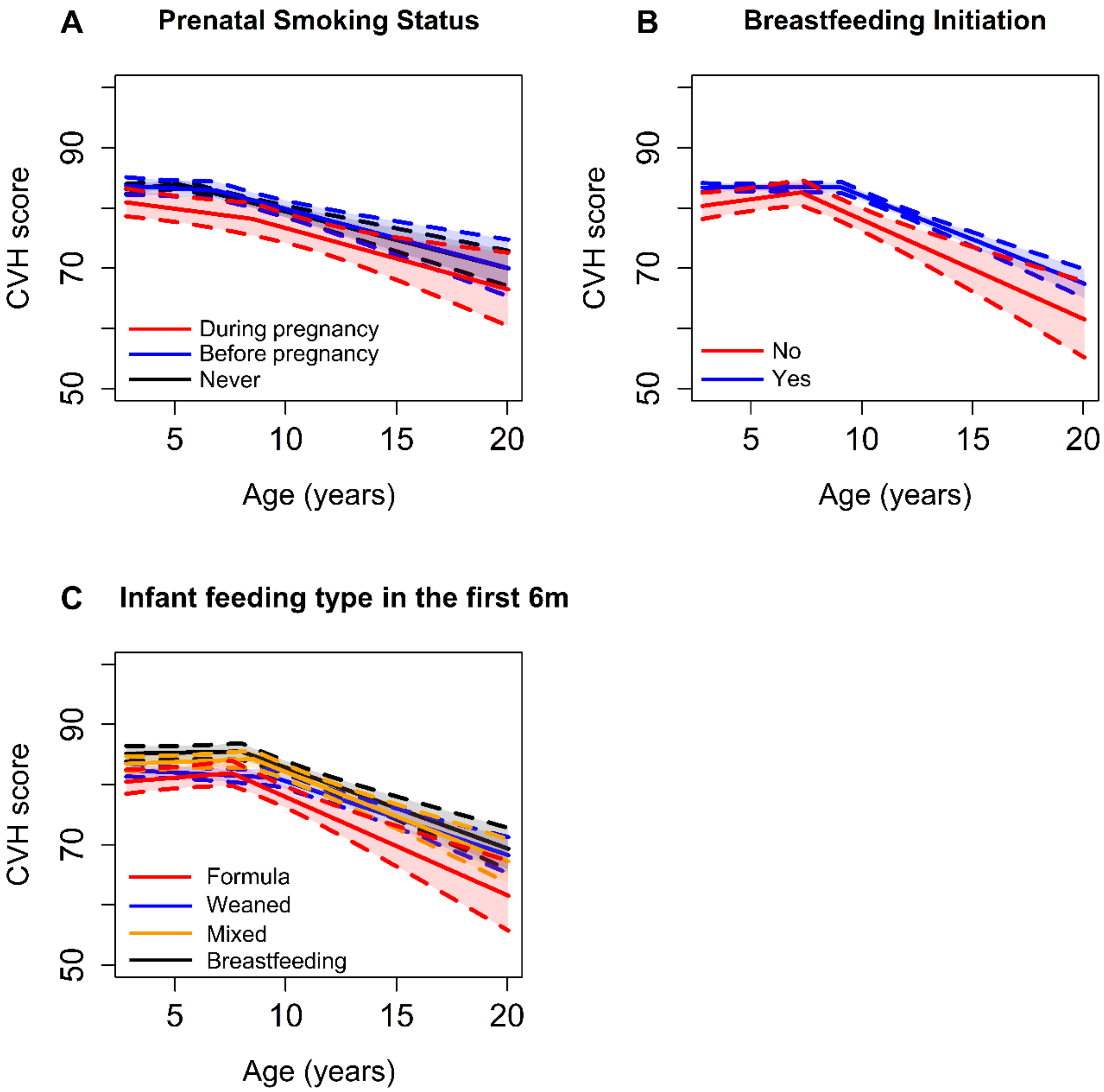

© 2025 Aris IM et al. *JAMA Network Open*.
