# Supplementary material for: Prenatal and Perinatal Factors of Life’s Essential 8 Cardiovascular Health Trajectories
Source: JAMA Netw Open. 2025 Apr 29;8(4):e257774. doi: 10.1001/jamanetworkopen.2025.7774 (PMC12042050; doi:10.1001/jamanetworkopen.2025.7774)
Supplement: Supplement 2. — Data Sharing Statement [file jamanetwopen-e257774-s002.pdf]

Data Sharing Statement

Aris. Pre- and Perinatal Factors of Life's Essential 8 Cardiovascular Health Trajectories. *JAMA Netw Open*. Published April 29, 2025. doi:10.1001/jamanetworkopen.2025.7774

Data
Data available: Yes
Data types: Deidentified participant data
How to access data: Data described in the article will be made available upon request pending application to the following email: VivaROADMaP@hphci.harvard.edu
When available: With publication

Supporting Documents
Document types: Statistical/analytic code
How to access documents: Code book and analytic code will be made available upon request pending application to the following email: VivaROADMaP@hphci.harvard.edu
When available: With publication

Additional Information
Who can access the data: Data will be made available to researchers whose proposed use of the data has been approved
Types of analyses: For a specified purpose
Mechanisms of data availability: After approval of an analytic proposal and a signed data access agreement
